# Molecular characterization of MRSA collected during national surveillance between 2008 and 2019 in the Netherlands

**DOI:** 10.1038/s43856-023-00348-z

**Published:** 2023-09-12

**Authors:** Leo M. Schouls, Sandra Witteveen, Marga van Santen-Verheuvel, Angela de Haan, Fabian Landman, Han van der Heide, Ed J. Kuijper, Daan W. Notermans, Thijs Bosch, Antoni P. A. Hendrickx, the Dutch MRSA surveillance study group, A. L. E. van Arkel, M. A. Leversteijn-van Hall, W. van den Bijllaardt, R. van Mansfeld, K. van Dijk, B. Zwart, B. M. W. Diederen, H. Berkhout, D. W. Notermans, A. Ott, K. Waar, W. Ang, J. da Silva, A. L. M. Vlek, A. G. M. Buiting, L. G. M. Bode, A. Jansz, S. Paltansing, A. J. van Griethuysen, J. R. Lo Ten Foe, M. J. C. A. van Trijp, M. Wong, A. E. Muller, M. P. M. van der Linden, M. van Rijn, S. B. Debast, E. Kolwijck, N. Al Naiemi, T. Schulin, S. Dinant, S. P. van Mens, D. C. Melles, J. W. T. Cohen Stuart, P. Gruteke, A. van Dam, I. Maat, B. Maraha, J. C. Sinnige, E. van der Vorm, M. P. A. van Meer, N. van Maarseveen, E. de Jong, S. J. Vainio, E. Heikens, M. den Reijer, J. W. Dorigo-Zetsma, A. Troelstra, E. Bathoorn, J. de Vries, D. W. van Dam, E. I. G. B. de Brauwer, R. Steingrover

**Affiliations:** 1 Centre for Infectious Disease Control. National Institute for Public Health and the Environment (RIVM), Bilthoven, The Netherlands; 2 Department of Medical Microbiology and Experimental Bacteriology, Leiden University Medical Center, Leiden, The Netherlands; 3 ADRZ medisch centrum, Department of Medical Microbiology, Goes, The Netherlands; 4 Alrijne Hospital, Department of Medical Microbiology, Leiden, The Netherlands; 5 Amphia Hospital, Microvida Laboratory for Microbiology, Breda, The Netherlands; 6 Amsterdam UMC—Location AMC, Department of Medical Microbiology and Infection Control, Amsterdam, The Netherlands; 7 Amsterdam UMC—Location Vumc, Department of Medical Microbiology and Infection Control, Amsterdam, The Netherlands; 8 Atalmedial, Department of Medical Microbiology, Amsterdam, The Netherlands; 9 Bravis Hospital/ZorgSaam Hospital Zeeuws-Vlaanderen, Department of Medical Microbiology, Roosendaal/Terneuzen, The Netherlands; 10 Canisius Wilhelmina Hospital, Department of Medical Microbiology and Infectious Diseases, Nijmegen, The Netherlands; 11 Centre for Infectious Disease Control, National Institute for Public Health and the Environment, Bilthoven, The Netherlands; 12 Certe, Medical Microbiology Groningen, Groningen, Drenthe The Netherlands; 13 Certe, Medical Microbiology Friesland | Noordoostpolder, Leeuwarden, The Netherlands; 14 Comicro, Department of Medical Microbiology, Hoorn, The Netherlands; 15 Deventer Hospital, Department of Medical Microbiology, Deventer, The Netherlands; 16 Diakonessenhuis Utrecht, Department of Medical Microbiology and Immunology, Utrecht, The Netherlands; 17 Elisabeth-TweeSteden (ETZ) Hospital, Department of Medical Microbiology and Immunology, Tilburg, The Netherlands; 18 Erasmus University Medical Center, Department of Medical Microbiology and Infectious Diseases, Rotterdam, The Netherlands; 19 Eurofins PAMM, Department of Medical Microbiology, Veldhoven, The Netherlands; 20 Franciscus Gasthuis & Vlietland, Department of Medical Microbiology and Infection Control, Rotterdam, The Netherlands; 21 Gelderse Vallei Hospital, Department of Medical Microbiology, Ede, The Netherlands; 22 Gelre Hospital, Department of Medical Microbiology and Infection Control, Apeldoorn, The Netherlands; 23 Groene Hart Hospital, Department of Medical Microbiology and Infection Prevention, Gouda, The Netherlands; 24 Haga Hospital, Department of Medical Microbiology, ‘s-Gravenhage, The Netherlands; 25 HMC Westeinde Hospital, Department of Medical Microbiology, ‘s-Gravenhage, The Netherlands; 26 IJsselland hospital, Department of Medical Microbiology, Capelle a/d IJssel, The Netherlands; 27 Ikazia Hospital, Department of Medical Microbiology, Rotterdam, The Netherlands; 28 Isala Hospital, Laboratory of Medical Microbiology and Infectious Diseases, Zwolle, The Netherlands; 29 Jeroen Bosch Hospital, Department of Medical Microbiology and Infection Control, ‘s-Hertogenbosch, The Netherlands; 30 LabMicTA, Regional Laboratory of Microbiology Twente Achterhoek, Hengelo, The Netherlands; 31 Laurentius Hospital, Department of Medical Microbiology, Roermond, The Netherlands; 32 Maasstad Hospital, Department of Medical Microbiology, Rotterdam, The Netherlands; 33 Maastricht University Medical Centre, Department of Medical Microbiology, Infectious Diseases & Infection Prevention, Maastricht, The Netherlands; 34 Meander Medical Center, Department of Medical Microbiology, Amersfoort, The Netherlands; 35 Noordwest Ziekenhuisgroep, Department of Medical Microbiology, Alkmaar, The Netherlands; 36 OLVG Lab BV, Department of Medical Microbiology, Amsterdam, The Netherlands; 37 Public Health Service, Public Health Laboratory, Amsterdam, The Netherlands; 38 Radboud University Medical Center, Department of Medical Microbiology, Nijmegen, The Netherlands; 39 Regional Laboratory for Microbiology, Department of Medical Microbiology, Dordrecht, The Netherlands; 40 Regional Laboratory of Public Health, Department of Medical Microbiology, Haarlem, The Netherlands; 41 Reinier de Graaf Groep, Department of Medical Microbiology, Delft, The Netherlands; 42 Rijnstate Hospital, Laboratory for Medical Microbiology and Immunology, Velp, The Netherlands; 43 Saltro Diagnostic Centre, Department of Medical Microbiology, Utrecht, The Netherlands; 44 Slingeland Hospital, Department of Medical Microbiology, Doetinchem, The Netherlands; 45 St Antonius Hospital, Department of Medical Microbiology and Immunology, Nieuwegein, The Netherlands; 46 St Jansdal Hospital, Department of Medical Microbiology, Harderwijk, The Netherlands; 47 Star-shl diagnostic centre, Department of Medical Microbiology, Rotterdam, The Netherlands; 48 TergooiMC, Central Bacteriology and Serology Laboratory, Hilversum, The Netherlands; 49 University Medical Center Utrecht, Department of Medical Microbiology, Utrecht, The Netherlands; 50 University of Groningen, Department of Medical Microbiology, Groningen, The Netherlands; 51 VieCuri Medical Center, Department of Medical Microbiology, Venlo, The Netherlands; 52 Zuyderland Medical Centre, Department of Medical Microbiology and Infection Control, Sittard, The Netherlands; 53 Zuyderland Medical Centre, Department of Medical Microbiology and Infection Control, Heerlen, The Netherlands; 54 St. Maarten Laboratory Services, Department of Medical Microbiology, Cay Hill (St. Maarten), St. Maarten

**Keywords:** Infectious-disease epidemiology, Epidemiology, Clinical microbiology, Antimicrobial resistance

## Abstract

**Background.:**

Although the Netherlands is a country with a low endemic level, methicillin-resistant *Staphylococcus aureus* (MRSA) poses a significant health care problem. Therefore, high coverage national MRSA surveillance has been in place since 1989. To monitor possible changes in the type-distribution and emergence of resistance and virulence, MRSA isolates are molecularly characterized.

**Methods.:**

All 43,321 isolates from 36,520 persons, collected 2008–2019, were typed by multiple-locus variable number tandem repeats analysis (MLVA) with simultaneous PCR detection of the *mecA*, *mecC* and *lukF-PV* genes, indicative for PVL. Next-generation sequencing data of 4991 isolates from 4798 persons were used for whole genome multi-locus sequence typing (wgMLST) and identification of resistance and virulence genes.

**Results.:**

We show temporal change in the molecular characteristics of the MRSA population with the proportion of PVL-positive isolates increasing from 15% in 2008–2010 to 25% in 2017–2019. In livestock-associated MRSA obtained from humans, PVL-positivity increases to 6% in 2017–2019 with isolates predominantly from regions with few pig farms. wgMLST reveals the presence of 35 genogroups with distinct resistance, virulence gene profiles and specimen origin. Typing shows prolonged persistent MRSA carriage with a mean carriage period of 407 days. There is a clear spatial and a weak temporal relationship between isolates that clustered in wgMLST, indicative for regional spread of MRSA strains.

**Conclusions.:**

Using molecular characterization, this exceptionally large study shows genomic changes in the MRSA population at the national level. It reveals waxing and waning of types and genogroups and an increasing proportion of PVL-positive MRSA.

## Plain language summary

A group of bacteria that cause difficult-to-treat infections in humans is methicillin-resistant *Staphylococcus aureus* (MRSA). The aim of this study was to monitor changes in the spread of MRSA, their disease causing potential and resistance to antibiotics used to treat MRSA infections. MRSA from patients and their contacts in the Netherlands were collected over a period of 12 years and characterized. This revealed new types of MRSA emerged and others disappeared. An increasing number of MRSA produces a protein called PVL toxin, enabling MRSA to cause more severe infections. Also, some people appear to carry MRSA without any disease for more than a year. These findings suggest an increasing disease potential of MRSA and possible unnoticed sources of infection. Consequently, it is important to maintain monitoring of these infections to minimize MRSA spread.

## Abstract (web summary)

Schouls et al. characterize 43,321 methicillin-resistant Staphylococcus aureus (MRSA) isolates obtained between 2008 and 2019 in the Netherlands. Genomic changes occur in the MRSA population, with increases in the proportion of PVL-positive MRSA.

## Introduction

The Netherlands is a country with a low endemic level of methicillin-resistant *Staphylococcus aureus* (MRSA). This is due to the restricted use of antibiotics and implementation of a so-called Search and Destroy policy, which relies on active screening of high-risk groups upon hospital admission, preventive isolation, and treatment of MRSA carriers [https://swab.nl/en/treatment-of-mrsa-carriage-general-information]^1^. Several studies have shown that the prevalence of MRSA carriage of patients admitted to Dutch hospitals is below 1%^2–4^. Nevertheless, MRSA may cause serious health problems, and changes in the prevalence and characteristics of MRSA should be closely monitored. Therefore, the Dutch national MRSA surveillance, in which all medical microbiology laboratories (MMLs) in the Netherlands send MRSA isolates to the National Institute for Public Health and the Environment (RIVM) for characterization, was started in 1989. The Dutch health inspectorate has urged the MMLs to submit MRSA isolates from all patients, and as a result, the coverage of the surveillance is high.

Several methods for the characterization of MRSA have been developed over the years, including spa-typing and a multiple-locus variable number of tandem repeat analysis (MLVA). However, classical multi-locus sequence typing (MLST), based on the sequence of seven housekeeping genes, has been widely used to identify MRSA lineages. The major lineages that have been found worldwide are clonal complexes CC1 (USA400), CC5 (USA100), CC8 (USA300), CC30 (USA200), and CC45 (USA600)^5–8^. Specific lineages have been linked to distinct types of infection, antibiotic resistance, and virulence. During the last decades, livestock has emerged as a major source of MRSA, colonizing and infecting humans in the Netherlands. These MRSA, designated as livestock-associated MRSA (LA-MRSA), belong to the MLVA-complex MC0389 (CC398) and currently makeup 25% of all isolates submitted in the surveillance. Several researchers have reported the low transmissibility and virulence of LA-MRSA. In other European countries and in North America, LA-MRSA is dominated by the clonal complex CC398, whereas in Asia, CC9 is the dominant LA-MRSA clonal complex^9^.

Here we report on the molecular characteristics and population structure of MRSA obtained from persons in Dutch healthcare centers and general practitioners (GPs). We use data from 43,321 MRSA isolates obtained from 36,250 persons, including 4991 sequenced isolates from 4798 persons collected in the 12-year surveillance period 2008–2019. The study reveals changes in the frequency in which MRSA lineages were found, distinct resistance and virulence gene profiles among the lineages, and reports on an increasing prevalence of MRSA isolates carrying genes encoding for Panton-Valentine Leukocidin (PVL), including LA-MRSA.

## Methods

### Bacterial isolates

Isolates were obtained for the Dutch national MRSA surveillance. All except one of the currently active 54 MMLs in the Netherlands send isolates from MRSA carriers and from persons infected with MRSA to the RIVM. All isolates were characterized by a multiple-locus variable number of tandem repeat analysis (MLVA) and simultaneous PCR detection of the *mecA, mecC*, and *lukF-PV* genes, the latter indicative of PVL production^10^. In the current study, isolates sampled from 2008–2019 that carried a *mecA* or *mecC* gene were typeable by MLVA and were obtained from persons with a person identifier were included. Isolates untypeable by MLVA were shown to be other species by MALDI-ToF analyses. Phenotypic antibiotic resistance data of the isolates and clinical data of the persons were not available. From 2008 to 2019, the Dutch MMLs submitted 43,321 isolates from 36,520 persons meeting these criteria (Supplementary Table 1). For 37,812 isolates from 32,020 persons, the genomic groups (GGs) were assessed either by whole genome multi-locus sequence typing (wgMLST) or inference of the GG based on the MLVA type. MRSA with MLVA types belonging to MLVA complex MC0398 (GG0398) were classified as LA-MRSA. The number of isolates used for the various analyses in this study differed, and an overview of these numbers is displayed in Supplementary Table 2. During the surveillance period, the MMLs submitted multiple isolates for some persons, and those included in the study are displayed in Supplementary Table 3.

### Metadata

All typing data and metadata were stored in the database of Type-Ned MRSA, a system for digital data exchange between the Dutch MMLs and the RIVM, which was rolled out nationwide in November 2016. All historical data from January 2008 until November 2016 were extracted from the RIVM laboratory information management system and imported into the Type-Ned database. The data comprised the patient’s gender, age in years (and in months if younger than two years), the four digits of the residential zip code, a pseudonymized person identifier (PID), and the specimen type. For isolates submitted via Type-Ned MRSA, the PID was based on the citizen service number, and for the historical data, the PID had been generated by an algorithm of the RIVM laboratory information management system. The distances between the residential geographic locations of the patients were assessed using the latitude and longitude of the 4-digit zip code acos(sin(lat1)*sin(lat2)+cos(lat1)*cos(lat2)*cos(lon2-lon1))*6371. To calculate the median distance and sampling intervals for genetic clusters of three or more isolates, pairwise distance, and sampling interval matrices for each isolate in a cluster were used.

### Next-generation sequencing and wgMLST

A subset of 4991 MRSA surveillance isolates from 4798 persons was subjected to next-generation sequencing (NGS) using the Illumina HiSeq 2500 (BaseClear, Leiden, the Netherlands) (Supplementary Table 1). Only isolates from persons for whom PIDs were available were sequenced. Virtually all isolates obtained in 2017–2019 from specimens other than swabs from the nose and/or throat and/or perineum (screening samples), all isolates from blood, and a randomly selected set of 25% of the isolates obtained from screening specimens were sequenced. In addition, all isolates (first isolate per person) obtained in the second quarter of 2019, irrespective of the specimen from which they originated, were included for sequencing. As a result, approximately 50% of the 2019 sequenced samples were from screening specimens. The remainder of the sequenced isolates (18%, 913/4491) was a convenience sample and originated from 2008 to 2016 surveillance isolates sequenced for various research questions. The NGS data were used for wgMLST analyses using the COL-based wgMLST scheme^11^ (2567 loci) and for classical MLST using SeqSphere software version 6.0.2 (Ridom GmbH, Münster, Germany). Both wgMLST and MLST profiles were imported into BioNumerics version 7.6.3 (Applied Maths, Sint-Martens-Latem, Belgium) and used in categorical clustering. Missing data were ignored in the analyses. Raw sequencing data of all Dutch MRSA surveillance isolates (2008–2022) have been deposited in the SRA database under BioProjects PRJNA880495, PRJNA881825, PRJNA880488, PRJNA882630, PRJNA884191, PRJNA891627, PRJNA893875, PRJNA897715, and PRJNA902678.

### Resistance and virulence

The presence of gene mutations predicted to lead to antibiotic resistance was assessed using the PointFinder software and databases from the Center for Genomic Epidemiology^12–15^. To identify acquired antibiotic resistance genes and virulence genes, reference gene sequences from the ResFinder and VirulenceFinder databases were used for BLAST analyses of assembled contigs obtained from the Illumina sequence data in the CLC Genomics Workbench software (Version 21.0.4, QIAGEN Aarhus A/S). Only hits yielding full-length genes (HSP length equals Query length) were considered to represent the gene. For an isolate that yielded a full-length yet imperfect hit (<100% identity), the sequence of the gene variant was extracted and classified as an NL-variant (NL=Netherlands) with nomenclature *gene*_NL_xx. As an example, *erm(C)*_NL_02 was the second sequence variant of the *erm(C)* gene that did not match with the *erm(C)* reference sequences in the ResFinder database. The nomenclature used for the gene variants present in the ResFinder (RF) and VirFinder (VF) databases was *gene*_RF_xx and *gene*_VF_xx. All gene variants were in silico and translated into proteins to assess whether they represented allelic variants of the genes. Gene variants with mutations leading to premature stop codons, thus yielding incomplete proteins, were excluded. The nomenclature used for the allelic variants was *gene*_Axxx. As an example, the gene variants *tet(K)*_RF_01 and *tet(K)*_NL_01 yielded the same allelic variant (*tet(K)*_A001) when translated. The sequences of all identified resistance and virulence genes and their allelic and gene variant assignments are provided in Supplementary Data 1 and Supplementary Data 2.

### Ethics statement

The bacterial isolates belong to the MMLs participating in the Dutch National MRSA Surveillance was obtained as part of routine clinical care in the past years. Only data on isolates and patients available in the digital Type-Ned system were used in this study. To ensure privacy, person identifiers were pseudonymized before storage in the Type-Ned database. Furthermore, only the patient’s age in years (not birthdate) and a residential region identifier based on the four digits of the zip code only were stored. Only MRSA isolates and not clinical specimens obtained from patients were available and used for this study. Since no identifiable personal data were collected and data were analyzed and processed anonymously, written or verbal patient consent was not required. According to the Dutch Medical Research Involving Human Subjects Act (WMO), this study was therefore exempt from review by an Institutional Review Board.

### Statistical analyses

Statistical analyses, such as two-sided Fisher’s exact test and two-tailed unpaired *t*-test, were performed in GraphPad version 9.4.0. Only results with a probability *p* < 0.05 were considered statistically significant. To calculate genotypic diversity, Simpson’s diversity index (DI = 1 − (∑*n*(*n* − 1)/*N*(*N* − 1)) was used.

### Reporting summary

Further information on research design is available in the Nature Portfolio Reporting Summary linked to this article.

## Results

### Characterization by MLVA

Since 2008 all isolates submitted for the Dutch MRSA surveillance have been typed by MLVA. Despite the 55% increase in the number of MLVA types for non-MC0398 isolates, from 935 MLVA types in 2008–2010 to 1447 in 2017–2019, the MLVA type-based diversity index did not change (Supplementary Table 5). Although the diversity index was high, the collection was dominated by a limited number of MLVA types, with 50% (*n* = 12,680) of the isolates carrying only 2% (61/3070) of all MLVA types (Supplementary Fig. 1). However, the frequency in which some MLVA types were found, changed considerably over time with a waxing and waning of types (Supplementary Fig. 2). For MC0398 (LA-MRSA) isolates, the number of MLVA types increased by 38% from 45 in 2008–2010 to 62 in 2017–2019, and the MLVA type-based diversity index increased from 0.619 to 0.700. Also, there was a considerable change in the composition of the MC0398 population. As an example, in 2008–2010, 3% (*n* = 68) of the MC0398 isolates had MLVA type MT569 which increased to 21% (*n* = 486) in 2017–2019.

### Characterization by wgMLST

**Fig. 1:**
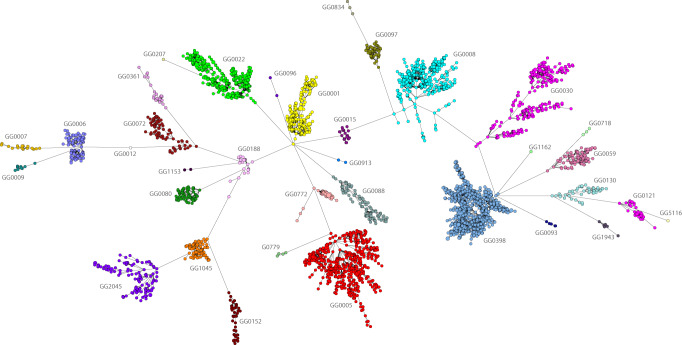
Population structure of MRSA in the Netherlands. The minimum spanning tree is based on categorical clustering of wgMLST data of 4798 MRSA isolates (first isolate per person). Each circle represents one or more MRSA isolates with the same wgMLST profile. Genogroups are indicated by colors and text. The distances between the genogroups, represented by the connecting lines, are at least 1000 wgMLST loci.

The NGS data of 4798 MRSA isolates (first isolate per person) were used to create wgMLST profiles which were subsequently used in categorical clustering. The profiles partitioned into 35 distinct groups differing from each other in at least 1000 of the 2567 wgMLST loci (Fig. 1). These genogroups (GGs) closely matched the classical MLST clonal complexes (CCs, BIGSdb) and were assigned similar names, e.g., GG0008 and CC8 (Supplementary Table 5, Supplementary Data 3). If a corresponding CC assignment was lacking in the BIGSdb, the dominant classical sequence type (ST) was used for the genomic group assignment, e.g., in GG0059, 57/106 are ST59. For some CCs, such as CC30, there was a perfect match with the GG. However, CC1 isolates were found among GG0001, GG0009, GG0188, GG0772, and GG0834. Forty percent (1917/4798) of the isolates had STs for which there was no CC assignment in the BIGSdb. The partitioning of the MRSA into GGs was retained in the minimum spanning trees obtained by classical MLST and MLVA (Supplementary Fig. 2). To compare the partitioning of Dutch isolates into GGs with that of *S. aureus* from other countries, we downloaded the 599 complete *S. aureus* chromosomes from the NCBI database that were available at that time, performed wgMLST and clustered the profiles together with the profiles of the 4798 MRSA isolates. This revealed that NCBI entries were partitioned in 24 of the 35 GGs (Supplementary Table 6). The Top10 GGs were also present among the NCBI entries, except for GG1045. There were 37 NCBI entries that were partitioned into 17 GGs that did not include isolates from the Dutch surveillance collection.

The average pairwise difference between the GGs was 1873 loci (Supplementary Fig. 4). GG0012–GG0718 and GG0718–GG0834 were the most distantly related GGs with a difference of 2042 and GG1045 and GG2045 were the most closely related GGS (1149 loci difference). The average difference between isolates within GGs varied considerably, from 396 loci for GG0030 to 60 loci for GG1045 (Supplementary Table 7). The highest intra-GG difference was found in GG0008 (928 loci), whereas GG1045 had the lowest intra-GG difference (249 loci).

An MLVA type always belonged to a single GG. This strict relationship allowed the assignment of hypothetical GGs based on the MLVA type for 32,725 of the 38,234 (86%) MRSA isolates for which no NGS data were available (Supplementary Table 1). This resulted in a collection of 37,812 isolates from 32,020 persons for which experimentally assessed or hypothetically inferred GGs which was used in the remainder of the study.

### Change during the prolonged carriage

For 14% of the persons (4496/32,020), multiple isolates that were sampled from the same person at different time points were submitted. The first and last sampled isolate of each person was used, and the sampling intervals for these pairs ranged from 0 days to 3079 days (8 years), with a mean of 407 days and a median of 173 days. Of the pairs that had identical GGs, 88% (3797/4327) also had identical MLVA types, and in 12% (*n* = 524), the MLVA profile of the second isolate was either a single locus or double locus variant (Supplementary Table 8). In only six persons, the pairs differed in three or four MLVA loci. In the pairs where the second isolate was a single locus variant, the most frequently changed MLVA locus (72%, 340/473) was V6, representing the protein A gene (spa). Except for one, all pairs with distinct GGs (*n* = 169) differed in at least three MLVA loci.

For 152 persons, multiple isolates were sequenced, allowing for a more in-depth analysis. Two persons from whom two isolates were sequenced, the first and last isolate belonging to distinct GGs, were excluded. For 128 persons, two isolates were sequenced, 74 carrying non-GG0398 MRSA and 54 carrying GG0398 MRSA. In only 5% (4/74) of the non-GG0398 pairs, the wgMLST profiles differed in more than 21 loci (Supplementary Data 4). In contrast, 41% (22/54) of the GG0398 pairs differed in more than 21 loci. This suggests that most persons carrying non-GG0398 isolates carried the same strain over a prolonged period, whereas a considerable number of persons carrying GG0398 MRSA acquired a new GG0398 strain. For the non-GG0398 isolates, the number of loci that differed between the isolates of a pair increased with increasing sampling interval (Supplementary Fig. 5). Probably due to the transient carriage, such a relationship was less clear for GG0398 pairs. For 22 persons, three or more sequenced isolates were available (Supplementary Data 4). In two of the 14 (14%) persons carrying non-GG0398 MRSA, one or more of the isolates differed in more than 21 loci. In contrast, five of the eight persons carrying GG0398 MRSA differed in more than 21 loci. In one person, two distinct GG0398 isolates (211 loci difference) were obtained on the same day. For the set of six isolates from a person (P032), the maximum pairwise distance between all six isolates was 13 loci involving 15 wgMLST loci. However, for another person (P003), the maximum pairwise distance was 26 loci, but there were 55 polymorphic wgMLST loci involved. Some loci seemed to change over time and remain the same thereafter, but other loci changed into a variant sequence and thereafter back into the original variant. Similar changes were observed even for isolates obtained on the same sampling day. These results strongly suggest that there is genetic heterogeneity of MRSA within the same person and that persons may carry more than one MRSA strain simultaneously.

### Geographic distribution of GGs

**Fig. 2:**
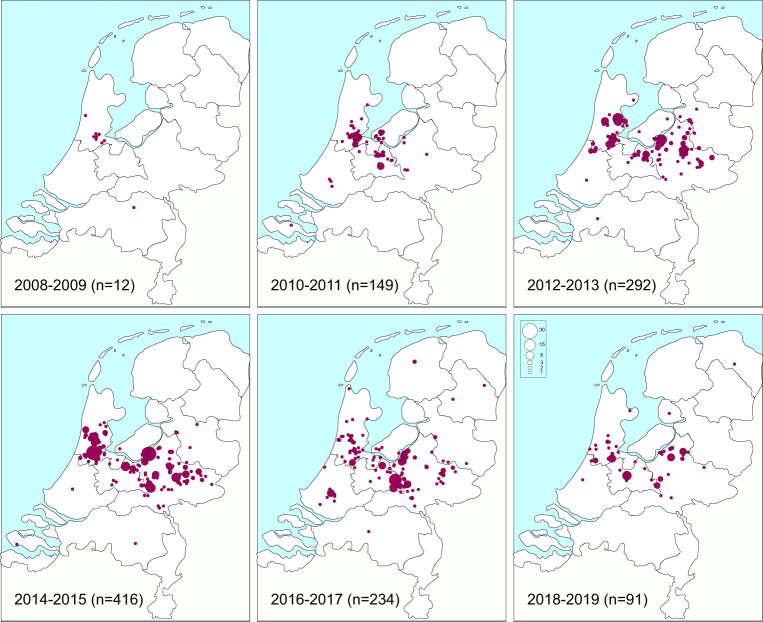
Temporal changes in the distribution of residential locations of persons from whom GG1045 were isolated (2008–2019, *n* = 1194, first isolate per person). The first isolates were sampled in the Mid-West of the country, after which GG1045 appeared to spread to the Mid-Eastern part of the country but not in the Northern or Southern direction.

All isolates with either experimentally or hypothetically assigned GGs were used to plot the residential location of persons carrying the various GGs. For most GGs, the geographic distribution followed the population density of the Netherlands, with most of the persons carrying the MRSA living in the most densely populated Mid-Western part of the country (Supplementary Fig. 6). There were two clear exceptions, people carrying GG0398 (LA-MRSA) and those carrying GG1045. As expected, approximately 90% of the persons with GG0398 lived in the Eastern part of the country, the region with intensive pig farming. The geographic distribution of residential locations of persons carrying GG1045 differed considerably from the other GGs. These locations were almost completely restricted to a region in the middle of the country from East to West. Only three GG1045 isolates were submitted in 2008; the annual submitted number of isolates increased thereafter, peaked in 2014 (*n* = 416), and decreased again in 2018–2019 (*n* = 91) (Fig. 2). The first isolates were obtained from persons living in the Western region where Amsterdam is located. Thereafter, the GG1045 MRSA seemed to spread toward the Eastern part of the country but remained mostly restricted to a strip in the middle of the country.

### Genetic clusters

The wgMLST profiles of isolates from persons for whom a 4-digit zip code was available were used in a categorical clustering. To prevent skewing because of unbalanced temporal distribution, only isolates obtained from 2017 to 2019 were used (first isolate per person, *n* = 3968). The analysis showed there were groups of closely related isolates within the GGs, referred to as genetic clusters. The number and size of these genetic clusters increased with the increasing number of different loci used to assign clusters, the cutoff. The range for the used cutoff was based on the change seen in persons with the persistent carriage. For most GGs, the number and cluster size remained unchanged at cutoffs higher than 21 loci, with, on average, 35% of the isolates partitioning into genetic clusters (example GG0008 in Supplementary Table 9). There were three exceptions: GG0121, GG0398, and GG1045. Sixty-eight percent of the GG0121 isolates partitioned in a single genetic cluster at the 7-loci-cutoff, increasing to 78% at the 35-loci-cutoff. Most of these isolates were part of an earlier described impetigo outbreak, which explains their close relationship^16^. For GG0398, the number of isolates that clustered kept increasing with an increasing cutoff. At the 21-loci-cutoff, 41% of all GG0398 isolates were partitioned into genetic clusters, with 37 isolates in the largest cluster, and the proportion of clustered isolates increased to 68% at the 35-loci-cutoff. For GG1045, 78% of the isolates clustered at the 21-loci-cutoff, with 34% of the isolates partitioning in the largest cluster. At the 35-loci-cutoff, the number of clusters was reduced to six, with 63% (40/56) of the clustered isolates in the largest cluster.

**Fig. 3:**
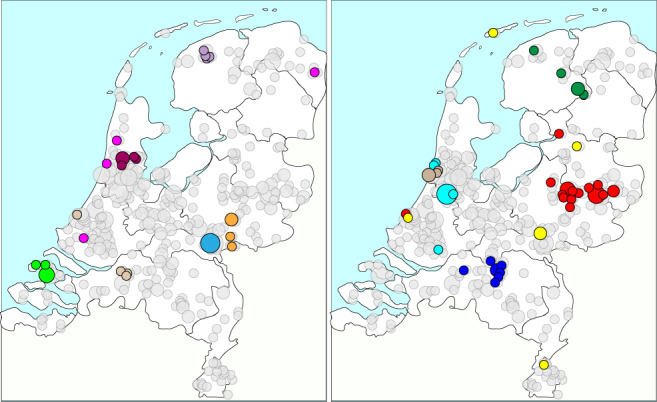
Plot of residential locations for the largest GG0005 genetic clusters on the map of the Netherlands. The genetic clusters were assigned using the 21-loci cutoff. Cluster isolates (first isolate per person) are displayed in color, and the light gray circles display all GG0005 isolates. The circle size represents the number of persons in the 4-digit zip code. The clusters have been displayed in two separate panels to prevent cluttering of the figure.

There was a clear spatial relationship of cluster isolates as illustrated by the plotting GG0005 genetic clusters on the map of the Netherlands (Fig. 3). For virtually all clusters, the residential locations of the persons from whom the isolates were obtained are close to each other. However, for some clusters, the fuchsia-colored and yellow clusters, locations were dispersed over the country.

At the 21-loci-cutoff, the 3968 isolates were grouped into 466 genetic clusters (Supplementary Table 10). The mutual distances of the residential locations for persons from whom isolates were partitioned into a genetic cluster were assessed. This resulted in an abundance of short distances with a median of 22 km among the 3598 distances (Supplementary Table 11, Supplementary Fig. 7). To exclude this distribution occurred by chance, a similar set of 3598 distances was created using randomly generated groups. For this set, the same 3968 isolates were used, and the number of groups and the group sizes per GG were chosen to be identical to those of the wgMLST genetic clusters. There was an almost Gaussian distribution of distances, and the median distance of 73 km of this random set was significantly higher than that of the genetic clusters, demonstrating the spatial character of genetic clusters. For the sampling interval, a similar approach was used (Supplementary Table 11, Supplementary Fig. 7). The median sampling interval for the isolates in the genetic clusters was 165 days and 240 days for the synthetic set. However, for genetic cluster distances lower or equal to the median distance of 22 km, the median sampling interval was only 84 days, suggesting combined spatial and temporal characteristics of genetic clusters.

### Predicted antibiotic resistance

The distribution of genes and mutations predicted to be leading to antibiotic resistance among the GGs was assessed using sequence data of isolates collected during the 2008–2019 surveillance period (*n* = 4798, one isolate per person). This revealed predicted resistance against 12 distinct antibiotic classes caused by 44 acquired and five mutated genes (Supplementary Table 12, Supplementary Data 1). The resistance level (*n*/N*100%) and the genes responsible for resistance differed between the GGs (Table 1). As an example, only 5% (13/250) of the GG0001 isolates were predicted to be ciprofloxacin resistant, whereas 98% of the GG1045 isolates carried mutations leading to ciprofloxacin resistance. Of the GG0398 isolates, 98% and only 2% of GG0022 isolates were resistant to tetracycline. Very few isolates (1%) were predicted to be rifampicin-resistant, and only 1% (9/1516) of the GG0398 were linezolid resistant. Many of the MRSA isolates were simultaneously resistant to multiple antibiotics (Supplementary Fig. 8 and Supplementary Table 13). GG1045 was the GG with the largest number of resistances, with 72% of the isolates resistant against at least three distinct antibiotics, and only a single isolate (1%) was sensitive to all nine antibiotics. In contrast, 53% of the GG0022 isolates were sensitive to all nine antibiotics, and only 10% were resistant to three antibiotics. Twenty-two percent (1055/4798) of all MRSA isolates were predicted to be sensitive to all nine antibiotics, whereas 28% were resistant to at least three of the nine antibiotics..

**Table 1:** The proportion (%) of isolates (first isolate per person) with predicted resistance against nine antibiotics due to acquired resistance genes or mutations among the GGs.

Antibiotic/gene	GG0001 (*n* = 250)	GG0005 (*n* = 620)	GG0008 (*n* = 554)	GG0022 (*n* = 507)	GG0030 (*n* = 284)	GG0059 (*n* = 106)	GG0080 (*n* = 69)	GG0398 (*n* = 1516)	GG1045 (*n* = 103)	GG2045 (*n* = 114)	Other GGs (*n* = 675)	All GGs (*n* = 4798)
Ciprofloxacin	5	42	44	42	17	8	6	22	98	36	23	30
* grlA, grlB, gyrA*^mut^	5	42	44	42	17	8	6	22	98	36	23	30
Clindamycin	65	27	22	21	10	71	29	41	28	12	14	30
*erm(A)*		15	3		7			11		9	0.4	7
*erm(B)*		0.5	0.2			68		6			4	4
*erm(C)*	65	12	18	21	3	5	29	16	28	4	10	17
*erm(T)*								3				1
*lnu(B)*								7			0.1	2
*lsa(A)*		0.2						0.5				0.2
*lsa(E)*								7			0.1	2
*vga(A)*								0.1				0.04
*vga(A)LC*								0.1				0.04
*vga(A)V*			1					3			0.4	1
*vga(E)*								9				3
Erythromycin	68	32	49	21	33	75	29	33	28	12	29	35
*erm(A)*		15	3		7			11		9	0.4	7
*erm(B)*		0.5	0.2			68		6			4	4
*erm(C)*	65	12	18	21	3	5	29	16	28	4	10	17
*erm(T)*								3				1
*mph(C)*	2	6	29		23	3					14	8
*msr(A)*	3	6	29	0.2	24	4		0.1			16	8
Fusidic acid	20	28	8	2	4	9	81	1	4	21	20	11
*fusA*^mut^		2	3	2	1	4		1	4	4	1	1
*fusB*		1	2		0.4	1	81			1		2
*fusC*	20	25	4	0.4	2	5		0.1		17	19	8
Gentamycin	18	4	11	16	4	6		19	73		25	16
*aac(6’)-aph(2”)*	18	4	11	16	4	6		18	73		22	15
*aph(2”)-Ia*		0.3	0.2					1			3	1
Linezolid								1				0.2
*cfr*								0.5				0.1
*optrA*								0.1				0.02
*poxtA*								0.1				0.02
Rifampicin		1	2	1	1	1			2	3	0.3	1
*rpoB*^mut^		1	2	1	1	1			2	3	0.3	1
Tetracycline	78	14	11	2	24	54	57	98	75	2	28	47
*tet(K)*	66	8	8	2	23	54	57	72	75	2	26	37
*tet(L)*	13	1	0.4		0.4			9			1	4
*tet(M)*	0.4	8	3		1			90	3		1	30
Trimethoprim	2	11	14	1	19			57	8	3	22	26
*dfrB*^mut^		1		1				0.1	7	3	3	1
*dfrD*								0.1			0.1	0.04
*dfrE*		0.3	0.2					1			3	1
*dfrG*	2	9	14	1	19			24	1		15	14
*dfrK*		0.5						33			1	11

Only the Top10 GGs are displayed. *N*, number of isolates per GG. n and %, number and percentage of resistant isolates per GG. Mut, mutations in the gene are responsible for the resistance. Only antibiotics relevant to the Dutch situation are displayed.

All isolates had been classified as either *mecA*- or *mecC*-positive based on the *mec*-PCR incorporated in the MLVA. However, no *mecA* or *mecC* genes were found in 12 sequenced isolates. Closer inspection revealed that nine contained incomplete *mecA* genes, and three carried no *mecA* or *mecC* genes, possibly due to loss during the subculture of the isolates in the laboratory. Among the 4730 isolates with an intact *mecA* gene, there were 76 *mecA* alleles with *mecA_A001* as the dominant allele present in 46% of the isolates (Supplementary Data 1). The was an association between GG and *mecA* allele. For example, *mecA_A001* was present in 84% of GG0008 but only in 3% of GG0080, in which 96% carried *mecA_A002*. The *mecA_A007* allele was found in 96% of GG1045 isolates but in only 4% of the GG0022 isolates. The *mecC* gene was found in 1% of the isolates, exclusively in all GG0130, GG1943, and GG5116 isolates (Supplementary Data 1). Most isolates (92%) carried *blaZ*, but *blaZ* was found in only 70% of the GG0006 isolates and none of the *mecC*-positive isolates.

There were many allelic variants of the acquired resistance genes (Supplemental Data 1). However, most genes were dominated by a single allele. The most genetically diverse gene was *blaZ*, for which 60 alleles were identified (DI = 0.863). However, only eight alleles comprised 90% of the *blaZ*-positive isolates. With 24 alleles, *fexA* was the second most diverse gene, but only eight alleles covered 90% of all *fexA*-positive isolates (DI = 0.761). The phenicol resistance gene *fexA* was found almost exclusively in GG0398 and GG0005^17^.

### Virulence factors

Genes predicted to encode virulence, such as immune evasion, toxins, and exoenzymes, were found among all GGs (Table 2, Supplementary Data 2). The arginine catabolic mobile element (ACME) was found in only 4% of the isolates. However, 24% of the GG0008 isolates carried this mobile element, making up 72% (133/185) of all ACME-positive isolates. The *scn* and *sak* host evasion genes are part of the phage-borne immune evasion cluster (IEC), which may also include either the *sea* or *sep* gene and the *chp* gene^18^. In silico IEC typing showed that the IEC type B, found in 35% of all isolates, was the dominant type (Supplementary Table 14). However, IEC-type distribution differed between GGs. For example, of GG0001 isolates, 60% belonged to IEC type E and 28% to IEC type D, 76% of GG0059 had IEC type C, and 98% of GG1045 were IEC type B. Of the 4798 isolates, 1290 (27%) did not carry any virulence gene other than the *aur* and *hlg* genes, and 1282 (99%) of these isolates belonged to GG0398, making up 85% of all GG0398 isolates. The *tst* gene encoding for the toxic shock syndrome toxin was found in low frequencies in eight of the Top10 GGs GGs. However, in GG0022, 62% of the isolates carried this toxin gene. Most isolates carried one or more enterotoxin genes, but the *seh* gene was found exclusively in 208 of the 209 GG0001 isolates.

**Table 2:** The proportion (%) of isolates (first isolate per person) with virulence genes per GG.

Virulence	Gene	GG0001 (*n* = 250)	GG0005 (*n* = 620)	GG0008 (*n* = 554)	GG0022 (*n* = 507)	GG0030 (*n* = 284)	GG0059 (*n* = 106)	GG0080 (*n* = 69)	GG0398 (*n* = 1516)	GG1045 (*n* = 103)	GG2045 (*n* = 114)	Other GGs (*n* = 675)	All GGs (*n* = 4798)
HostImmunity	ACME	0.4	5	24	1	1			0.1		11	0.3	4
	*sak*	90	83	87	89	99	22	96	12	98	90	75	61
	*scn*	91	83	87	89	99	98	97	12	98	90	84	64
Toxin	*edinA*		6									0.3	1
	*edinB*							99				1	2
	*edinC*											4	1
	*eta*								0.1			6	1
	*etb*											4	1
	*hlgA*	99.6	100	99.8	47	99	99	100	99.7	100	98	99	94
	*hlgB*	99	99.5	99	100	100	99	100	99.9	100	100	99	99.5
	*hlgC*	100	99	98	99	99.6	100	97	99.7	87	95	99	99
	*lukD*	99.6	98	95				100				84	42
	*lukE*	98	88	91				97				79	40
	*lukF-PV*	17	19	70	20	78	66	83	7			25	27
	*lukS-PV*	17	19	70	22	78	66	83	7			25	27
	*tst*	2	10	3	62	16			0.1	1	4	1	10
Exoenzyme	*aur*	99	97	97	99.8	99.6	100	100	99.9	100	98	99	99
	*splA*	99	100	82				100				72	39
	*splB*	98	99	84				100				101	43
	*splE*	29		82		88						49	23
Enterotoxin	*sea*	29	5	8	7	7	6		0.4		5	24	8
	*seb*	2	5	3			86	3	3		4	4	5
	*sec*	2	6	0.2	24	1				1	39	16	7
	*seg*		100		99	99				95	97	24	37
	*seh*	99.6											5
	*sei*		98		99	98				96	99	30	38
	*sej*		49	23						89	2	0.3	11
	*sek*	28	0.5	62		0.4	84	3	0.2		6	2	11
	*sel*	2	6	0.2	24	1			0.2	1	46	10	6
	*sem*		98		97	97				97	100	30	37
	*sen*		99		98	100				90	95	29	37
	*seo*		99		99	98				38	100	30	36
	*sep*	0.4	43	1		0.4	5		0.3			8	7
	*seq*	29	0.5	60		0.4	83	3	0.2		6	2	11
	*ser*		46	22						89	2	0.1	11
	*seu*					99.6						7	7

Only the results of Top10 GGs are displayed in detail. *N*, number of isolates per GG. *n* and %, number and percentage of isolates with virulence factor per GG.

The PVL encoding *lukF-PV* and *lukS-PV* gene pair were found in eight of the Top10 GGs but not in GG1045 and GG2045. For some PVL-positive isolates, the assembly resulted in contigs carrying complete PVL bacteriophage genomes allowing the identification of the type of phage (Supplementary Table 15). Seventy-six percent of the PVL-positive GG0005 isolates and all PVL-positive GG0121 isolates carried the *lukF-PV* and *lukS-PV* genes on truncated phage genomes (Supplementary Fig. 9). The two types of PVL-positive GG0005 isolates were present in distinct branches in the wgMLST minimum spanning tree (Supplementary Fig. 10). All 111 GG0398 PVL-positive isolates carried phage genomes that were virtually identical to ϕSa2wa_st93 (GenBank acc.nr. MG029517). In all isolates, the phage genomes were flanked by the attachment sequence (*attR*), and they were inserted into the same chromosomal region.

### Temporal changes in GGs

**Fig. 4:**
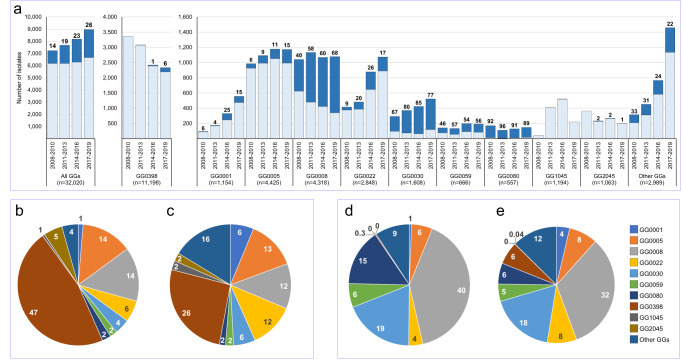
Temporal changes in the composition of the MRSA population. **a** The stacked dark blue bars represent the PVL-positive, and the light blue bars the PVL-negative isolates. The numbers above the bars denote the PVL-positive proportion (%) of the isolates. Pie charts of the distribution of all isolates in 2008–2010 (**b**) and 2017–2019 (**c**). Distribution of the PVL-positive isolates in 2008–2010 (**d**) and 2017–2019 (**e**). The numbers in and just outside the pie chart segments denote the proportions (%) of each GG.

The assignment of hypothetical GGs allowed analyses of changes during the 12-year surveillance period using 32,020 isolates (first isolate per person). This showed there was an increase in the number of annually submitted MRSA isolates from 2270 in 2008 to 2989 in 2019 (32%) (Supplementary Table 1). There also was an increase in the proportion of submitted PVL-positive isolates from 14% in 2008–2010 to 26% in 2017–2019 (Fig. 4, Supplementary Table 16). For GG0005, both the number of submitted isolates and the proportion of PVL-positive isolates increased. In contrast, the number of submitted GG0008 remained the same, yet the proportion of PVL-positive isolates increased from 40% in 2008–2010 to 68% in 2017–2019. The number of submitted GG0398 decreased considerably during the surveillance period decreasing from 3365/7225 (47% of all isolates) in 2008–2010 to 2335/8952 (26% of all isolates) in 2017–2019. The overall composition of the population remained similar, although some GGs, such as GG0001 and GG0022 isolates, were submitted considerably more frequently in recent years.

### Relationship of specimens and GGs

There were associations between the GG and the specimen type from which the MRSA was cultured (Supplementary Fig. 11). Although isolates obtained from sputum and from screening swabs (from nose/throat/perineum) were dominated by GG0398, virtually all the other GGs were also found in these specimen types. GG0008 was the dominant GG among MRSA obtained from pus and wound, and these were mostly PVL-positive isolates (73–87%). The PVL-encoding genes were the only putative virulence genes where there was a relationship with specimen type. Bloodstream infections with MRSA are rare in the Netherlands, and among the 32,020 isolates in the collection, only 287 (0.9%) were isolated from blood. Of interest was the observation that GG0398 was the most frequently found GG (20%, 57/287) obtained from blood. However, only 0.5% of all GG0398 isolates were obtained from blood, which is slightly, yet significantly lower than for most other GGs (0.4–1.4%). As GG0398 makes up 35% of the collection, sheer numbers may have caused the contribution of GG0398 from blood. Nevertheless, it demonstrates its potential to cause severe disease. There was no clear association between the specimen type and antibiotic resistance except for ciprofloxacin resistance. Approximately 30% of the isolates were resistant to ciprofloxacin. However, 56% of MRSA obtained from urine were ciprofloxacin resistant. With approximately 70%, the proportion of ciprofloxacin-resistant isolates from urine was considerably higher than in isolates obtained from specimens other than urine (30–40%).

### PVL in GG0398

**Fig. 5:**
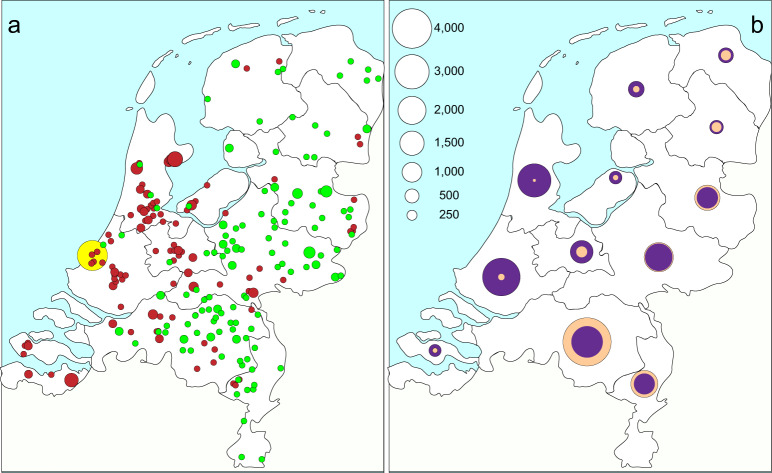
Different geographic distribution of the residential location in the Netherlands of persons carrying PVL-positive and PVL-negative GG0398 MRSA. **a** All 2017–2019 PVL-positive GG0398 MRSA (red circles, *n* = 140) and a random sample of PVL-negative GG0398 (green circles, *n* = 140) from the same period. The circle size indicates the number of sampled persons living in the same geographic location. The yellow circle on the West Coast denotes the city of The Hague. **b** The number of inhabitants of the 12 Dutch provinces (purple) and the number of pigs in pig farms per province (salmon). The circle sizes represent the number of inhabitants or pigs per province ×1000. In the province in the Mid-East (Gelderland), the number of inhabitants is nearly the same as the number of pigs.

Although the marked decrease in the number of submitted GG0398 isolates over time, its contribution to the set of all PVL-positive isolates increased from 0.3% in 2008–2010 (*n* = 3) to 6% in 2017–2019 (*n* = 141) (Fig. 4). The distribution of the residential locations of persons carrying PVL-positive GG0398 MRSA differed considerably from those with PVL-negative GG0398 (Fig. 5a). The separation between PVL-positive and PVL-negatives is not absolute, but the mean distance of the residential locations of the persons with PVL-positive GG0398 to the city of The Hague on the West coast of the Netherlands was 62 km, whereas those of persons carrying PVL-negative GG0398 was 99 km (Supplementary Table 17). Considering the Netherlands is only 315 km North to South and 265 km East to West, a difference of 37 km is considerable. Statistically, significantly more persons (*t*-test, *p* < 0.0001) carrying PVL-positive GG0398 were found in Western part of the country, where population density is highest and the number of pigs in pig farms the lowest (Fig. 5b). For other GGs, PVL-positive isolates were also more frequently found in the Western part of the country, although only for GG0008 with a significant F test (variances, *p* < 0.0001).

**Fig. 6:**
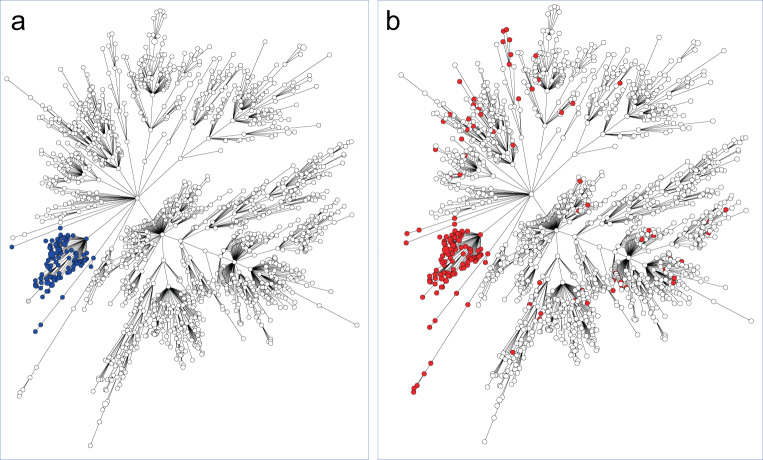
wgMLST minimum spanning trees of 1516 GG0398 isolates (2008–2019, first isolate per person). **a** 111 isolates carrying *lukS-PV/lukF-PV* are in dark blue. **b** 181 isolates carrying *sak/scn* genes are in red.

The PVL-positive GG0398 isolates were found in three separate branches in the wgMLST minimum spanning tree (Fig. 6). The two smaller branches comprised the oldest isolates, sampled in 2010–2014, and these all had ST398. In the large branch, 95/104 isolates had ST1232, and none had ST398. Remarkably all PVL-positive GG0398 isolates carried the *sak* and *scn* genes, but these genes were present in only 5% (70/1405) of the PVL-negative GG0398. The PVL-positive and PVL-negative GG0398 isolates had a distinct composition of the resistome (Supplementary Table 18). Of interest is the fact that none of the PVL-positive isolates carried the *tet(M)* gene, generally considered to indicate animal origin. In contrast, 97% of the PVL-negative GG0398 isolates carried this gene. Another noticeable difference was the presence of the aminoglycoside resistance gene *ant(9)-Ia*, found in 93% of the PVL-positive isolates compared to 12% in the PVL-negative GG0398 isolates. Also, the allelic variant of *ant(9)-Ia* differed between the two groups. This was also the case for the *blaZ* gene, with 93 % of the PVL-positive isolates carrying *blaZ_A007* and only 0.1% in the PVL-negatives where *blaZ_A216* (72%) dominated. None of the PVL-positive isolates carried *tet(M)*, whereas 97% of the PVL-negatives carried this tetracycline resistance gene.

## Discussion

Here we describe the molecular characteristics and population structure of MRSA in the Netherlands based on the characterization of 37,812 isolates obtained from 32,020 persons during 12 years of national MRSA surveillance. The surveillance has very high nationwide coverage of participating medical microbiology laboratories submitting MRSA isolates obtained from infected patients and asymptomatic carriers. To our knowledge, this is the largest study reported on the molecular epidemiology of MRSA in a single country over a long period of time.

The MRSA population was composed of 35 wgMLST GGs, and the 10 most frequently found GGs made up 91% of all isolates, as reported before^19–22^. The largest GG was GG0398 comprising 36% of the isolates, followed by GG0005 (14%) and GG0008 (13%). During the 12-year surveillance period, the frequency in which the GGs were found changed, but this did not result in an increased MRSA population diversity. Comparison with the epidemiology in other European countries is hampered by the lack of molecular data obtained from national surveillance programs. Most studies are regional, cover a short period, and are on invasive diseases only. Four Nordic countries performed a joint MRSA surveillance (2009–2016)^23^. They demonstrated a temporal increase of CC6 and CC22 and a decrease of CC8, which is consistent with the changes seen in the Netherlands. In Denmark, CC398 increased considerably, and t034/CC398 made up 34% of all isolates in 2014. In the Netherlands, GG0398 also was the predominant GG, but in contrast to Denmark, there was a decrease after 2010. The proportion of PVL-positive MRSA has been increasing in the Netherlands from 15% to 25% during the study period. Similar increases have also been reported in other countries^23,24^.

The geographic distribution of residential locations of persons in the Netherlands was similar for most GGs reflecting the population density, except for GG1045 and GG0398. For GG1045, distribution changed over time, with occurrence starting in the Mid-West in 2008 and gradual movement to the Mid-East thereafter. Of the GG1045 isolates, 98% had spa-type t1081, and two previous studies linked this GG to transmission between long-term care facilities via healthcare workers^25,26^. In the study presented here, 98% of the GG1045 isolates carried mutations predicted to lead to ciprofloxacin. Such a high proportion would fit with the high levels of ciprofloxacin usage in nursing homes. At its peak in 2015, 19% of all MRSA isolates were GG1045, and in 2019 this was reduced to 3% of all MRSA. In 2022 GG2045 was nearly completely gone, with only two GG0245 isolates submitted for the Dutch MRSA surveillance.

In 95% of the cases, the wgMLST profiles obtained from sets of multiple non-GG0398 isolates from the same person differed in ≤21 wgMLST loci from each other. Therefore, the 21-loci-cutoff was used to identify genetic clusters in the collection. The median distance of the residential location of persons whose isolates were partitioning in genetic clusters was 22 km, and for 33% of the persons, the distance was 10 km or less. The sampling interval of isolates within a genetic cluster was relatively high, with a median of 165 days. This shows a clear spatial but only a weak temporal character of the genetic clusters. Cutoff values that are utilized to assess transmission are often the source for debate, and several papers have described such cut-offs^27,28^. It is unclear from this study if the 21-loci-cutoff can be used in transmission studies because no data on possible contact and transmission between persons were available. Also, this cutoff might be unsuitable for some GGs, such as GG1045 and GG0398, which have only limited genetic diversity. However, the data show that if isolates belong to the same genetic cluster, it often demonstrates regional spread within a limited time.

There were clear relationships between the GG and in silico resistance profiles for nine antibiotics used in the Netherlands to treat persons for MRSA carriage or infection. In GG0398, 52% of the isolates simultaneously carried genes associated with resistance to more than two of these antibiotics, and only 1% did not carry genes inducing resistance to these antibiotics. This makes GG0398 the second most multi-resistant GG after GG1045. In contrast, 53% of GG0022 MRSA lacked resistance genes for any of the nine antibiotics and only 10% against more than two antibiotics. From this study, it is unclear what role the various putative virulence factors play, as most GGs carry multiple genes that have been implicated in the virulence of MRSA. In GG0398, 84% of the sequenced isolates did not carry any virulence gene, excluding *aur* and *hlg*. The proportion of isolates carrying the *lukF-PV* gene was much higher in MRSA cultured from pus and wounds than those obtained from urine, sputum, and screening swabs. This seems to corroborate that PVL, indeed is an important virulence factor which is especially evident from its association with skin and soft tissue infections. Overall, there was a considerable increase in PVL-positivity, rising from 14% in 2008–2010 to 26% in 2017–2019. It is unclear what has caused this increase. Possibly, PVL-positive MRSA causes more severe disease motivating MMLs to submit these isolates. Alternatively, infections caused by PVL-positive MRSA may result in increased bacterial load on the skin and therefore be more transmissible than PVL-negative MRSA.

The lack of phenotypic resistance profiles, treatment, clinical data, and outcome of disease were important limitations of this study. The phenotypic consequences of the presence of genes and mutations in genes predicted to lead to antibiotic resistance could not be assessed. Furthermore, the lack of data on antibiotic treatment regimens and the success and failure of treatment made evaluation of the consequences of putative resistance impossible. The lack of clinical data, such as the severity and duration of the disease, has hampered the interpretation of the relevance of the putative virulence genes for diseases caused by MRSA. Also, we were unable to classify isolates as ‘clinical’ or ‘screening,’ and such properties could only be conjectured from the specimen types from which the MRSA were cultured. Collection of both phenotypic antibiotic resistance data and proper clinical data in future MRSA surveillance would be highly recommendable. Furthermore, the availability of epidemiological data on suspected transmission due to contact between persons may help to identify types, GGs, and mechanisms responsible for enhanced transmissibility.

Only 7% of the GG0398 isolates were cultured from blood, pus, and wounds suggesting lower virulence, which is in line with earlier findings^29,30^. However, due to the high number of GG0398 isolates in the collection, it ranks second in the proportion of isolates obtained from blood, pus, and wounds. GG0398 in the Netherlands has acquired PVL, and in 2017–2019 PVL-positivity had increased to 6%. Forty-three percent of the PVL-positive isolates were found in blood, pus, and wounds, underpinning the importance of PVL for virulence. GG0398 is mostly considered relatively harmless with hampered transmission capabilities, but this study suggests otherwise^31,32^. The PVL-positive GG0398 isolates were predominantly found in the densely populated Western part of the Netherlands, where the number of pig farms is low. Possibly human-to-human transfer of the is depending on population density and not on contact with livestock. This would imply that we may see an accelerated increase of PVL-positive GG0398, and we should closely monitor this development. The PVL-positive GG0398 comprised a distinct subclade in the wgMLST tree with ST1232, suggesting that one or more strains have picked up a PVL-bacteriophage that is virtually identical to the ϕSa2wa_st93. One of these strains is now either evolving or the phage is being transferred to other closely related strains. All sequenced PVL-positive GG0398 isolates also carried the ϕSa3 phage carrying *sak* and *scn* genes. Recently, Japanese researchers reported two patients from whom they isolated PVL-positive CC398 isolates with ST1232 that carried the *sak* and *chp* genes^33,34^. Shortly thereafter, an ST1232 isolate from a Korean patient was studied in a detailed comparison with the isolates from Japan^35^.

The wgMLST GGs closely match the classical seven-gene-based MLST clonal complexes. However, in the list of profiles and STs provided by the BIGSdb, 33% of the STs do not have a CC assigned^36^. In literature, CCs are often introduced without apparent reference to a repository or the origin of this classification. The wgMLST GG assignment, based on a 1000 loci difference between GGs, is a much more robust approach for international standardized nomenclature of groups of genetically related *S. aureus* and is backward compatible with classical MLST. Although MLVA and MLST can be used to some extent for surveillance, these methods are outdated and should be replaced by whole-genome sequencing. This allows for accurate genetic grouping and clustering of MRSA and enables the study of other characteristics, such as the mechanisms of antibiotic resistance and virulence. It would also be prudent to add long-read third-generation sequencing to the analyses to better elucidate the impact of mobile elements such as plasmids, transposons, and bacteriophages which play major roles in the horizontal transfer of antibiotic resistance and virulence genes.

### Supplementary information

Supplementary Data 1

Supplementary Data 2

Supplementary Data 3

Supplementary Data 4

Supplemental Information

Reporting Summary

Description of Additional Supplementary Files

## Data Availability

All raw sequence data are available in the Sequence Read Archive (see Methods). Supplementary Data 1 contains the distribution of all resistance gene variants among the GGs, including their DNA sequences. Supplementary Data 2 contains the distribution of all virulence gene variants among the GGs, including their DNA sequences. Supplementary Data 3 displays the relationship between GGs, MLVA types, CCs, and MLST types for 4798 sequenced isolates of the study. Supplementary Data 4 contains the genetic distances between multiple MRSA isolates obtained from 152 persons. The numerical data underlying Fig. 4 is in Supplementary Table 16. Other data are available from the last author Antoni Hendrickx (Antoni.Hendrickx@rivm.nl), on reasonable request.
