# Supplementary material for: Molecular characterization of MRSA collected during national surveillance between 2008 and 2019 in the Netherlands
Source: Commun Med (Lond). 2023 Sep 12;3:123. doi: 10.1038/s43856-023-00348-z (PMC10497500; doi:10.1038/s43856-023-00348-z)
Supplement: Supplementary file 5 — Supplemental Information [file 43856_2023_348_MOESM5_ESM.pdf]

Supplementary Table 1

Number of isolates and persons from whom these isolates were obtained.

	All with PID		GGExp		GGHyp		GGExp or GGHyp		No GGExp or GGHyp	
Sampling year	Persons	Isolates	Persons	Isolates	Persons	Isolates	Persons	Isolates	Persons	Isolates
2008	2,685	2,948	39	47	2,241	2,430	2,270	2,477	434	471
2009	2,827	3,361	59	65	2,399	2,812	2,447	2,877	399	484
2010	2,951	3,540	61	73	2,467	2,928	2,508	3,001	469	539
2011	2,913	3,525	76	96	2,471	2,956	2,522	3,052	414	473
2012	2,918	3,605	63	84	2,482	3,023	2,526	3,107	425	498
2013	3,040	3,773	22	35	2,611	3,208	2,620	3,243	457	530
2014	2,858	3,569	50	52	2,381	2,970	2,422	3,022	481	547
2015	3,163	3,772	97	100	2,654	3,142	2,740	3,242	451	530
2016	3,468	3,918	358	361	2,703	3,029	3,013	3,390	479	528
2017	3,430	3,951	1,213	1,225	2,062	2,346	3,155	3,619	297	332
2018	3,053	3,534	1,082	1,114	1,870	2,094	2,808	3,240	259	294
2019	3,214	3,825	1,678	1,739	1,552	1,787	2,989	3,542	251	283
Total	36,520	43,321	4,798	4,991	27,893	32,725	32,020	37,812	4,816	5,509

PID, person identifier. GGExp, experimentally assessed GG, i.e. based on wgMLST using sequence data. GGHyp, hypothetical GG inferred from the MLVA type.

Supplementary Table 2

Number of isolates and persons used in the various analyses described in the Results section.

Results section	Sampling years	Persons	Isolates	Remarks
Characterization by MLVA	2008-2019	36,520	36,520	First isolate per person
Characterization by wgMLST	2008-2019	4,798	4,798	First sequenced isolate per person
Prolonged carriage, MLVA	2008-2019	4,496	8,992	Persons with multple isolates, frst and last isolates per person only
Prolonged carriage, sequencing	2008-2019	152	345	Persons with multple sequenced isolates, all isolates
Genetic clusters	2017-2019	3,968	3,968	First sequenced isolate per person (2017-2019)
Geographic distribution of GGs	2008-2019	29,451	29,451	Persons with zip code, first isolate per person
Predicted antibiotic resistance	2008-2019	4,798	4,798	First sequenced isolate per person
Virulence factors	2008-2019	4,798	4,798	First sequenced isolate per person
Temporal changes in genogroups	2008-2019	32,020	32,020	Isolates with GGexp or GGhyp, first isolate per person
Relationship of specimens and GGs	2008-2019	32,020	32,020	Isolates with GGexp or GGhyp, first isolate per person
PVL in GG0398, MLVA	2008-2019	11,196	11,196	GG0398 isolates (GGExp or GGHyp), first isolate per person
PVL in GG0398, sequencing	2017-2019	1,516	1,516	GG0398 isolates (GGExp), first isolate per person (2017-2019)

Supplementary Table 3

Number of multiple isolates per person with PIDs, submitted by the MML during 2008-2019.

	MLVA				NGS			
	Persons		Isolates		Persons		Isolates	
Isolates per person	*n*	%	*n*	%	*n*	%	*n*	%
1	27,524	86	27,524	73	4,646	97	4,646	93
2	3,572	11	7,144	19	130	3	260	5
3	699	2	2,097	6	14	0.3	42	1
4	142	0.44	568	2	3	0.1	12	0.2
5	51	0.16	255	1	3	0.1	15	0.3
6	15	0.05	90	0.2	1	0.02	6	0.1
7	9	0.03	63	0.2				
8	4	0.01	32	0.1				
9	2	0.01	18	0.05				
10	1	0.003	10	0.03	1	0.02	10	0.2
11	1	0.003	11	0.03				
Total	32,020		37,812		4,798		4,991	

Supplementary Table 4

Temporal changes in the number and distribution of MLVA types in 36,520 isolates (first isolate per person).

MRSA group	Period	Num. MLVA types	Num. isolates	DI
Non-MC0398	2008-2010	935	5,109	0.988
	2011-2013	1,131	5,805	0.987
	2014-2016	1,324	7,080	0.990
	2017-2019	1,447	7,367	0.993
MC0398	2008-2010	45	3,354	0.619
	2011-2013	44	3,066	0.657
	2014-2016	46	2,409	0.657
	2017-2019	62	2,330	0.700

DI, Simpson's diversity index.

Supplementary Figure 1

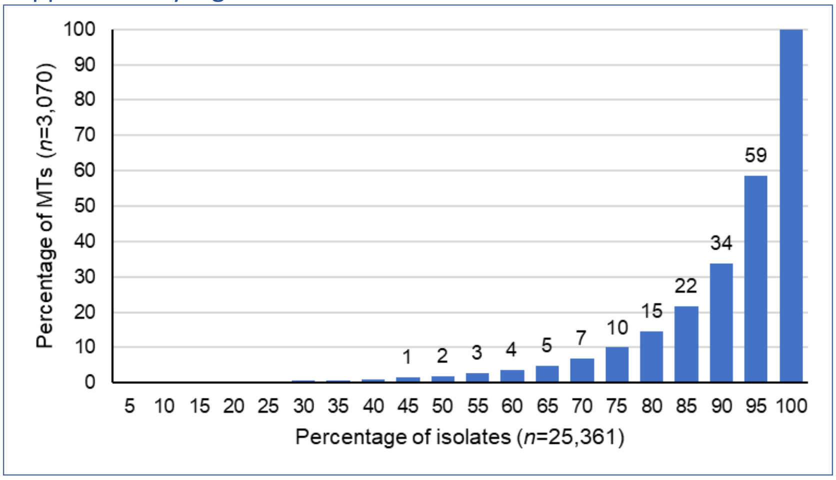

MLVA-based diversity of the non-MC0398 isolates (first isolate per person). *MLVA types were first sorted by frequency after which the proportion of isolates was plotted against the proportion of MLVA types. The numbers above the bars indicate the proportion of MLVA types, e.g., 10% (n=306) of the MLVA types made up 75% (n=19,019) of the isolates.*

Supplementary Figure 2

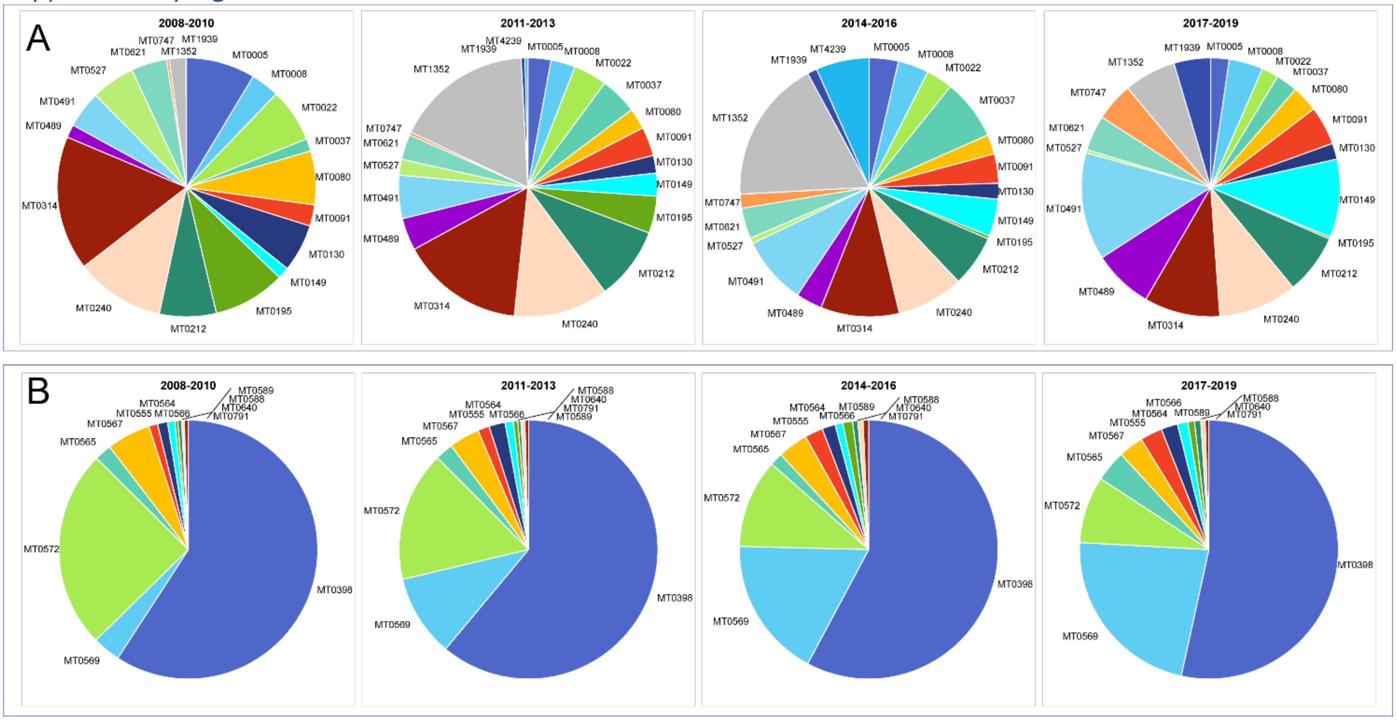

Pie charts showing the temporal change in the frequency in which predominant MLVA types were found (first isolate per person). *Panel A displays pie charts for non-MC0398 and panel B for MC0398. The total number of types in the charts panel A is 20 MVLA types and in panel B 12 MLVA types. These are the MLVA types that belonged to the 10 most frequently found types in each of the four time periods.*

Supplementary Table 5

Relationship between wgMLST GGs and MLST CCs of sequenced isolates (2008-2019, n=4,798, first isolate per person).

	CC assignment based on BigsDB database												
GG	CC1	CC5	CC8	CC15	CC22	CC30	CC45	CC93	CC97	CC121	No CC	No ST	Total
GG0001	250												250
GG0005		620											620
GG0006		128											128
GG0007			14								3		17
GG0008			549									5	554
GG0009	10												10
GG0012											1		1
GG0015				26									26
GG0022					506						1		507
GG0030						284							284
GG0059											106		106
GG0072			72								5		77
GG0080											69		69
GG0088											88		88
GG0093								8					8
GG0096											2		2
GG0097									68				68
GG0121										41			41
GG0130											49		49
GG0152											43		43
GG0188	25												25
GG0207											1		1
GG0361											23		23
GG0398					1					1	1,510	4	1,516
GG0718											3		3
GG0772	46												46
GG0779											4		4
GG0834	3												3
GG0913											2		2
GG1045							103						103
GG1153									2				2
GG1162											1		1
GG1943											6		6
GG2045							114						114
GG5116										1			1
Total	334	748	635	26	507	284	217	8	70	43	1,917	9	4,798

CC assignment was obtained from the BIGSdb database. No CC, no CC assigned for the ST identified. No ST, the MLST profile was either incomplete or not yet assigned.

Supplementary Figure 3

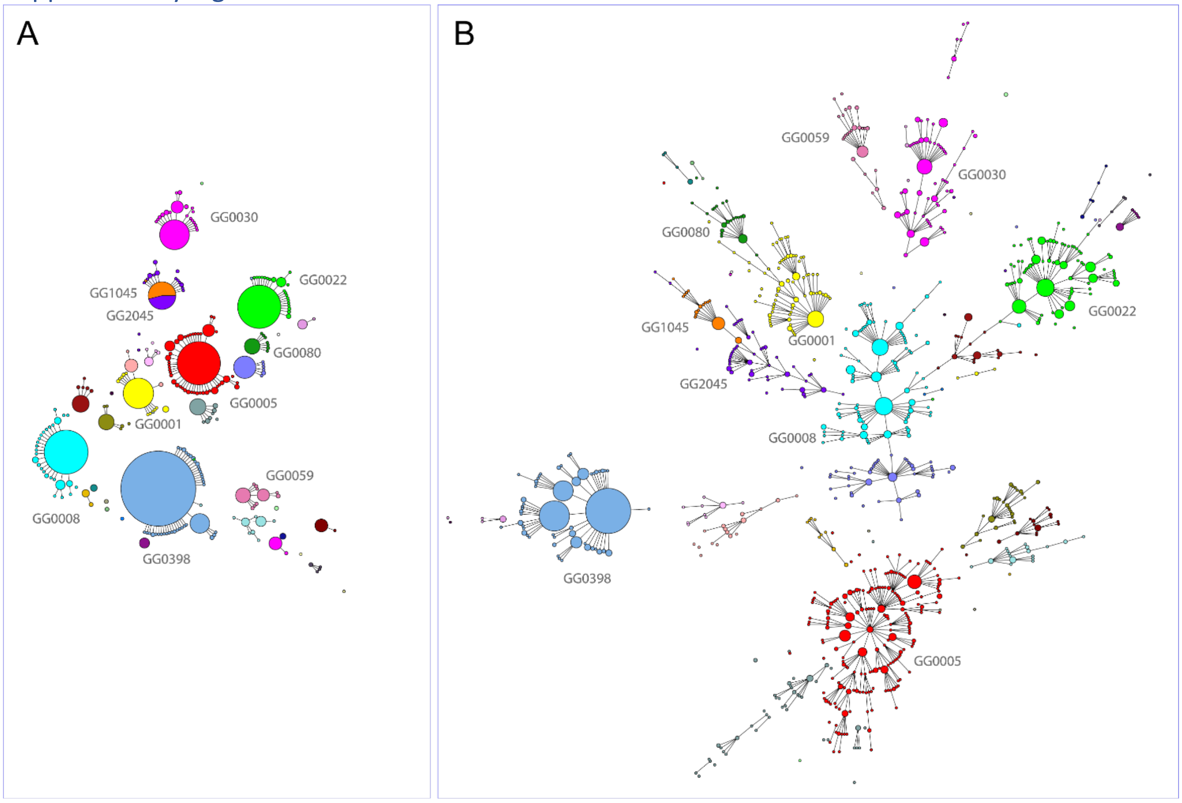

Minimum spanning trees using MLST data (A) and MLVA data (B) of 4,798 sequenced isolates (first isolate per person). *The colors of the circles represent the GGs and are identical to those in Figure 1 of the main text. Only the GGs assignments for the Top10 GGs are shown as text in the figures. Sequence types and MLVA types that differ in a single locus are connected by a line.*

Supplementary Table 6

Presence of genogroups among 599 complete *S. aureus* chromosomes found in the NCBI database.

GG	*N*	Num. NCBI entries	NCBI Reference
GG0001	250	28	NC_002953.3
GG0005	620	125	NC_002745.2
GG0008	554	183	NC_002951.2
GG0022	507	14	NZ_CP007659.1
GG0030	284	48	NC_002952.2
GG0059	106	20	NC_016928.2
GG0080	69	4	NC_017351.1
GG0398	1,516	45	NZ_CP013218.1
GG1045	103		
GG2045	114	19	NC_021554.1
GG0006	128	3	NZ_CP047021.1
GG0007	17	5	NZ_CP015646.1
GG0009	10	20	NZ_CP011528.1
GG0012	1	1	NZ_CP041037.1
GG0015	26	17	NZ_CP012970.1
GG0072	77	12	NC_022226.1
GG0088	88	4	NZ_CP019563.1
GG0093	8	1	NC_017338.2
GG0096	2		
GG0097	68	14	NZ_LR134087.1
GG0121	41	12	NZ_CP013137.1
GG0130	49	7	NZ_CP025395.1
GG0152	43	3	NZ_CP024998.1
GG0188	25	6	NZ_AP018922.1
GG0207	1		
GG0361	23		
GG0718	3		
GG0772	46	7	NZ_CP010526.1
GG0779	4		
GG0834	3		
GG0913	2		
GG1153	2	1	NZ_CP065857.1
GG1162	1		
GG1943	6		
GG5116	1		
All GGs	4,798	599	

N, number of isolates per GG in the surveillance collection. The last column in the table displays the NCBI accession number of one of the entries (NCBI reference sequence) that partitioned in the GG.

Supplementary Figure 4

	n	GG0001	GG0005	GG0006	GG0007	GG0008	GG0009	GG0012	GG0015	GG0022	GG0030	GG0059	GG0072	GG0080	GG0088	GG0093	GG0096	GG0097	GG0121	GG0130	GG0152	GG0188	GG0207	GG0361	GG0398	GG0718	GG0772	GG0779	GG0834	GG0913	GG1045	GG1153	GG1162	GG1943	GG2045
GG0001	248																																		
GG0005	612	1727																																	
GG0006	127	1665	1729																																
GG0007	17	1682	1727	1353																															
GG0008	545	1612	1720	1702	1700																														
GG0009	10	1635	1681	1615	1669	1685																													
GG0012	1	1697	1719	1614	1649	1687	1643																												
GG0015	26	1604	1723	1673	1687	1595	1640	1637																											
GG0022	502	1849	1877	1901	1893	1879	1875	1911	1897																										
GG0030	284	1917	1949	1956	1956	1747	1937	1974	1967	1946																									
GG0059	106	1943	1984	1999	1986	1952	1976	2002	1996	1984	1939																								
GG0072	77	1652	1719	1614	1649	1675	1643	1585	1680	1911	1957	1994																							
GG0080	69	1611	1737	1692	1715	1621	1683	1689	1655	1881	1957	1979	1627																						
GG0088	87	1697	1709	1715	1732	1713	1734	1743	1717	1899	1955	1997	1729	1737																					
GG0093	8	1944	1969	1995	1985	1975	1982	2006	1987	1961	1913	1906	1989	1979	1987																				
GG0096	2	1671	1752	1720	1715	1731	1700	1725	1707	1917	1973	2003	1700	1713	1780	2015																			
GG0097	68	1665	1732	1715	1707	1609	1634	1732	1663	1879	1957	1987	1697	1630	1715	1976	1751																		
GG0121	39	1949	1995	2001	1994	1979	1991	2012	1992	1965	1912	1941	1993	1997	1992	1917	2007	1999																	
GG0130	47	1945	1987	1989	1977	1974	1963	1996	1989	1966	1903	1927	1982	1989	1995	1893	1991	1985	1664																
GG0152	42	1936	1979	1996	1983	1975	1991	1991	1972	1964	1919	1974	1991	1995	1995	1966	1983	1979	1974	1961															
GG0188	23	1451	1707	1650	1672	1627	1623	1637	1667	1879	1935	1967	1601	1605	1720	1959	1700	1635	1976	1961	1961														
GG0207	1	1909	1936	1955	1947	1934	1944	1979	1962	1815	1951	1985	1949	1952	1959	1983	1993	1953	1973	1975	1971	1934													
GG0361	23	1707	1734	1661	1687	1707	1674	1635	1724	1922	1973	2007	1609	1675	1760	1989	1733	1714	2009	2007	1997	1673	1961												
GG0398	1,018	1870	1914	1917	1921	1905	1909	1936	1919	1913	1813	1911	1932	1917	1929	1897	1936	1917	1902	1887	1934	1911	1931	1932											
GG0718	3	1986	2007	2033	2013	2005	1997	2042	2017	1993	1929	1681	2024	2022	2017	1942	2039	2019	1949	1945	1987	2002	2011	2024	1917										
GG0772	45	1603	1577	1697	1707	1679	1670	1687	1657	1903	1967	2007	1683	1654	1723	1977	1732	1625	1995	1991	1991	1653	1973	1689	1924	2024									
GG0779	4	1745	1605	1753	1743	1722	1723	1753	1757	1916	1959	2001	1753	1741	1772	1989	1763	1763	2002	1985	2006	1753	1953	1754	1939	2031	1750								
GG0834	3	1655	1737	1730	1727	1654	1650	1755	1694	1914	1969	2006	1727	1713	1755	2009	1752	1400	2006	1986	1989	1699	1974	1732	1934	2042	1657	1767							
GG0913	2	1563	1767	1707	1735	1653	1703	1729	1645	1917	1969	2009	1675	1641	1751	2007	1687	1684	1993	1997	1999	1615	1982	1719	1943	2041	1610	1772	1714						
GG1045	100	1907	1956	1969	1961	1933	1953	1976	1959	1939	1883	1925	1964	1953	1973	1909	1972	1964	1926	1917	1917	1555	1946	1982	1867	1936	1959	1979	1971	1967					
GG1153	2	1735	1781	1767	1754	1785	1784	1772	1767	1913	1964	1991	1731	1749	1794	2009	1773	1789	2007	1997	2017	1723	1977	1775	1959	2034	1762	1795	1795	1782	1974				
GG1162	1	1961	1969	1992	1989	1986	1972	2023	1994	1977	1897	1937	1993	2002	1995	1949	2017	1981	1914	1896	1965	1982	1991	2009	1894	1982	2001	1999	2005	2023	1917	2006			
GG1943	4	1976	2005	2023	2004	1995	2003	2036	2017	1984	1923	1939	2017	2013	2014	1935	2034	2017	1777	1772	1975	2002	1985	2026	1919	1969	2023	2019	2041	2037	1923	2029	1929		
GG2045	107	1903	1947	1969	1964	1926	1947	1971	1954	1937	1872	1933	1961	1949	1966	1914	1964	1961	1921	1909	1924	1797	1949	1975	1871	1941	1955	1973	1969	1965	1149	1967	1917	1921	
GG5116	1	1977	1997	2004	1999	2001	1982	2033	1996	1997	1922	1929	2007	2005	2001	1922	2027	2004	1699	1703	1977	2006	2003	2019	1909	1957	2013	2009	2021	2023	1935	2026	1937	1820	1924

Pairwise distances between the wgMLST GGs among 4,798 MRSA isolates (first isolate per person). *The salmon-coloured cells denote the GGs with largest number of different loci. The green-coloured cells denote the GGs with the smallest number of different loci. n, the number of isolates per GG.*

Supplementary Table 7

Number of different loci within the genogroups among 4,798 MRSA isolates (first isolate per person).

		Number of different loci			
GG	*N*	Average	Median	Minimum	Maximum
GG0001	250	214	143	0	477
GG0005	620	324	331	0	499
GG0006	128	123	111	1	269
GG0007	17	138	106	1	265
GG0008	554	316	285	1	928
GG0009	10	88	51	8	288
GG0015	26	49	53	3	91
GG0022	507	262	318	0	403
GG0030	284	396	41	0	657
GG0059	106	192	163	0	393
GG0072	77	171	173	0	337
GG0080	69	149	148	1	327
GG0088	88	260	297	1	365
GG0093	8	73	82	1	98
GG0096	2	563	563	563	563
GG0097	68	139	151	1	208
GG0121	41	283	329	0	615
GG0130	49	236	300	1	372
GG0152	43	127	133	0	225
GG0188	25	158	105	1	665
GG0361	23	143	117	5	313
GG0398	1,516	176	191	0	352
GG0718	3	47	45	43	53
GG0772	46	200	120	0	735
GG0779	4	103	119	3	141
GG0834	3	130	130	123	137
GG0913	2	163	163	163	163
GG1045	103	60	53	0	249
GG1153	2	106	106	106	106
GG1943	6	317	318	106	527
GG2045	114	369	391	0	540

N, number of isolates per GG. GG012, GG0207, GG1162 and GG5116 each comprised a single isolate and were omitted from the table.

Supplementary Table 8

Differences in MLVA profiles after putative persistent MRSA carriage in isolates from 4,496 persons from whom multiple isolates were obtained.

MLVA loci		Same GG		Distinct GGs	
different	*N*	*n*	%	*n*	%
0	3,797	3,797	88		
1	473	473	11		
2	52	51	1	1	1
3	7	5	0.1	2	1
4	11	1	0.02	10	6
5	27			27	16
6	48			48	28
7	61			61	36
8	20			20	12
Total	4,496	4,327		169	

N, total number of persons with multiple isolates. n and %, number and percentage of isolates with same or distinct GG.

Supplementary Figure 5

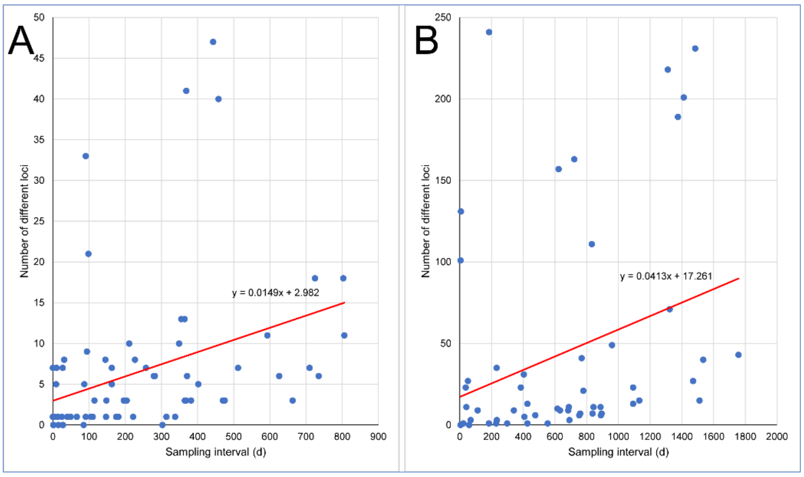

Relationship between the number changed loci and sampling interval of pairs of isolates obtained from the same person. *A, isolates pairs from 74 persons carrying non-GG0398 MRSA and B, isolates pairs from 54 persons carrying GG0398 MRSA. A linear trendline with its equation is displayed in each panel in red. Note that the scales of the X- and Y-axes in the panels are different.*

Supplementary Figure 6

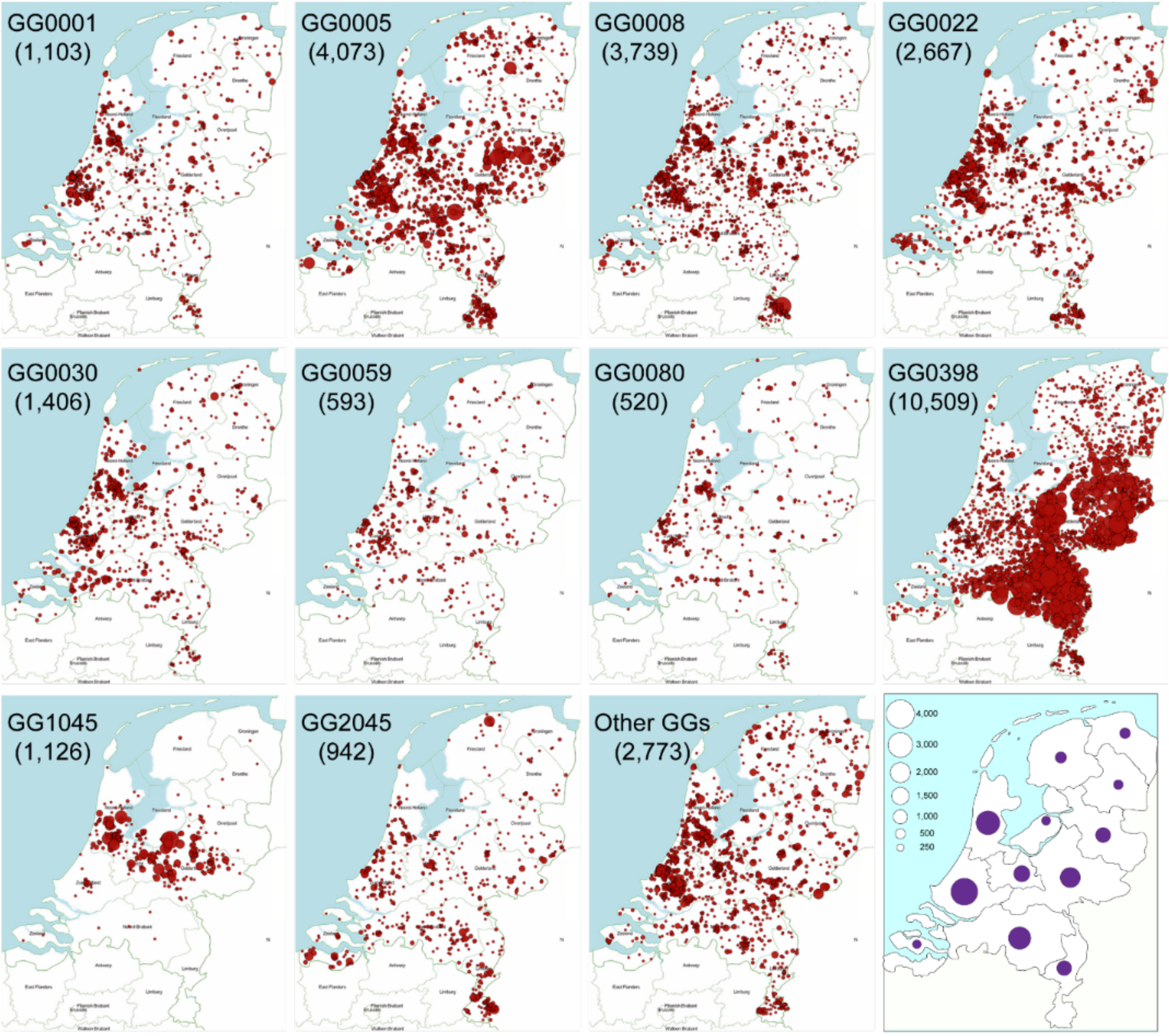

Geographic distribution of residential locations of persons carrying MRSA (n=29,451 isolates, 2008-2019, first isolate per person). *The numbers in parenthesis are the number of persons carrying MRSA with the GG. The sizes of the circles represent the number of persons living in the zip code area. The lower right panel displays the population density in the Netherlands with the circle size indicating the number of persons x1,000.*

Supplementary Table 9

Relationship between genetic cluster size, number of clusters and the cutoff used for assigning clusters for five GGs.

		7-loci-cutoff				14-loci-cutoff				21-loci-cutoff				28-loci-cutoff				35-loci-cutoff				42-loci-cutoff			
GG	*N*	Size	Num	*n*	%	Size	Num	*n*	%	Size	Num	*n*	%	Size	Num	*n*	%	Size	Num	*n*	%	Size	Num	*n*	%
GG0008	440	2	18	36	8	2	30	60	14	2	28	56	13	2	28	56	13	2	27	54	12	2	26	52	12
		3	4	12	3	3	10	30	7	3	8	24	5	3	10	30	7	3	12	36	8	3	11	33	8
		4	1	4	1	4	1	4	1	4	4	16	4	4	7	28	6	4	6	24	5	4	6	24	5
		6	3	18	4	5	1	5	1	5	1	5	1	5	1	5	1	5	2	10	2	5	4	20	5
						7	1	7	2	6	1	6	1	6	1	6	1	6	1	6	1	6	1	6	1
						8	1	8	2	7	2	14	3	7	1	7	2	7	1	7	2	7	2	14	3
						9	1	9	2	8	1	8	2	8	2	16	4	8	2	16	4	8	2	16	4
										9	1	9	2	9	1	9	2	9	1	9	2	9	1	9	2
			26	70	16		45	123	28		46	138	31		51	157	36		52	162	37		53	174	40
GG0121	40	2	2	4	10	27	1	27	68	27	1	27	68	2	2	4	10	2	2	4	10	6	1	6	15
		23	1	23	58									27	1	27	68	27	1	27	68	27	1	27	68
			3	27	68		1	27	68		1	27	68		3	31	78		3	31	78		2	33	83
GG0398	1,163	2	58	116	10	2	94	188	16	2	87	174	15	2	74	148	13	2	51	102	9	2	31	62	5
		3	8	24	2	3	18	54	5	3	24	72	6	3	26	78	7	3	22	66	6	3	14	42	4
		4	5	20	2	4	13	52	4	4	12	48	4	4	8	32	3	4	8	32	3	4	7	28	2
		5	2	10	1	5	3	15	1	5	5	25	2	5	6	30	3	5	7	35	3	5	4	20	2
		11	1	11	1	8	1	8	1	6	3	18	2	6	2	12	1	6	2	12	1	6	3	18	2
						11	1	11	1	7	2	14	1	8	2	16	1	7	1	7	1	7	1	7	1
										8	4	32	3	9	2	18	2	8	1	8	1	8	3	24	2
										9	2	18	2	11	2	22	2	9	1	9	1	9	2	18	2
										10	2	20	2	13	1	13	1	11	1	11	1	11	2	22	2
										11	1	11	1	16	1	16	1	17	1	17	1	16	1	16	1
										12	1	12	1	17	1	17	1	19	1	19	2	18	1	18	2
										37	1	37	3	18	1	18	2	20	1	20	2	43	1	43	4
														39	1	39	3	24	1	24	2	61	1	61	5
														42	1	42	4	35	1	35	3	65	1	65	6
														46	1	46	4	45	1	45	4	66	1	66	6
														99	1	99	9	56	1	56	5	103	1	103	9
																		73	1	73	6	106	1	106	9
																		85	1	85	7	168	1	168	14
																		132	1	132	11				
			74	181	16		130	328	28		144	481	41		130	646	56		104	788	68		76	887	76
GG1045	64	2	5	10	16	2	2	4	6	2	3	6	9	2	3	6	9	2	3	6	9	2	1	2	3
		3	2	6	9	3	4	12	19	3	3	9	14	3	1	3	5	3	1	3	5	14	1	14	22
		4	1	4	6	4	3	12	19	4	3	12	19	4	1	4	6	7	1	7	11	43	1	43	67
		13	1	13	20	13	1	13	20	10	1	10	16	7	1	7	11	40	1	40	63				
										13	1	13	20	10	1	10	16								
														22	1	22	34								
			9	33	52		10	41	64		11	37	78		8	20	81		6	56	88		3	59	92

*N, total number of isolates per GG. Size, the number of isolates within a genetic cluster. Num, number of clusters with the indicated cluster size. n, number of isolates in cluster size class. %, percentage of all isolates per GG (n/N*100).*

Supplementary Table 10

Number of genetic clusters per cluster size at the 21-loci-cutoff in the Top10 GGs.

GG	GG0001		GG0005		GG0008		GG0022		GG0030		GG0059		GG0080		GG0398		GG1045		GG2045		Other GGs		All GGs	
N	241		511		440		432		258		94		66		1,163		64		94		605		3,968	
Cluster size	Clust	Isol	Clust	Isol	Clust	Isol	Clust	Isol	Clust	Isol	Clust	Isol	Clust	Isol	Clust	Isol	Clust	Isol	Clust	Isol	Clust	Isol	Clust	Isol
2	14	28	39	78	28	56	29	58	28	56	6	12	6	12	87	174	3	6	7	14	36	72	283	566
3	6	18	15	45	8	24			7	21	3	9	2	6	24	72	3	9	2	6	11	33	90	270
4	3	12	5	20	4	16	9	27	1	4					12	48	3	12	3	12	7	28	38	152
5	1	5	4	20	1	5	2	10							5	25					2	10	15	75
6			1	6	1	6	3	18							3	18					1	6	9	54
7			1	7	2	14	1	7							2	14							6	42
8	1	8			1	8									4	32			1	8	1	8	8	64
9			1	9	1	9									2	18							4	36
10															2	20	1	10					3	30
11							1	11							1	11					1	11	3	33
12															1	12							1	12
13																	1	13					1	13
17							1	17															1	17
21							1	21															1	21
22			1	22																			1	22
27																					1	27	1	27
37															1	37							1	37
Total	25	71	67	207	46	138	47	169	36	81	9	21	8	18	144	481	11	50	13	40	60	195	466	1,471

Clust, number of clusters; Isol, number of isolates.

Supplementary Table 11

Spatial (distance between residential location) and temporal (sampling interval) properties of genetic clusters.

			Percentile		
	**Cluster/group**	**Mean**	**25%**	**50%**	**75%**
Distance (km)	Genetic clusters	40	7	22	57
	Random groups	81	39	73	117
Sampling interval (days)	Genetic clusters	262	36	165	436
	Random groups	291	87	240	450

Supplementary Figure 7

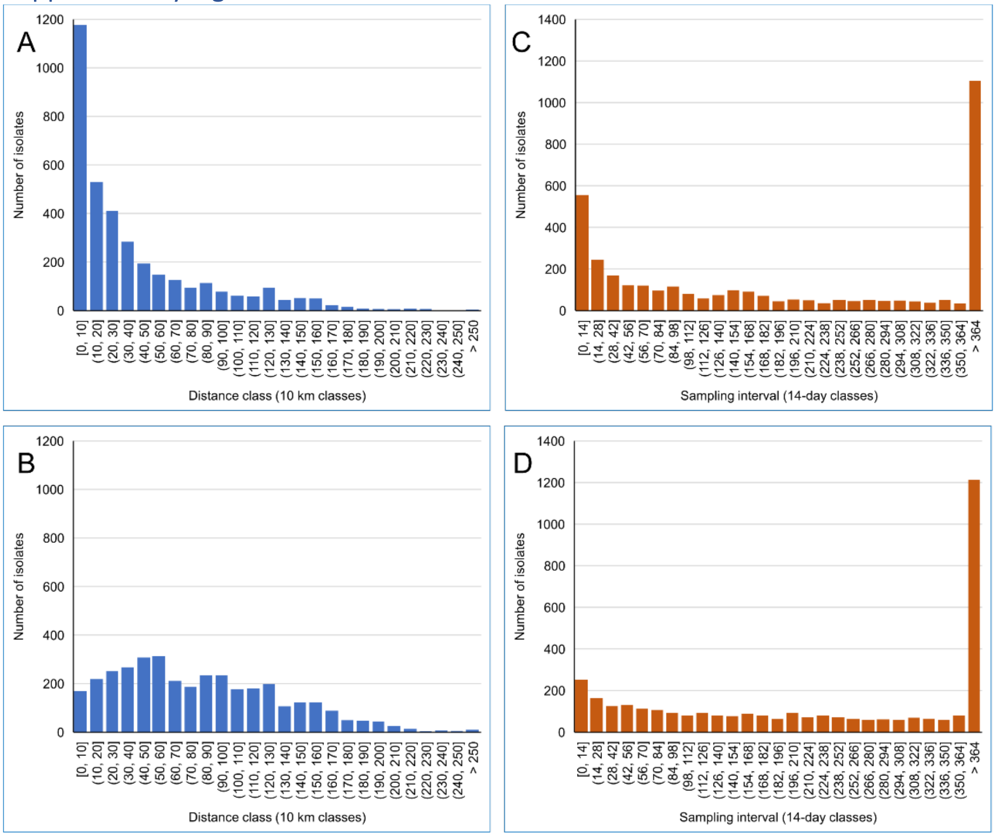

Histograms of spatial (distance between residential location) and temporal (sampling interval) properties of genetic clusters. *A, number of isolates in genetic clusters within the distance classes; B, number of isolates in synthetic groups within the distance classes; C, number of isolates in genetic clusters within the sampling interval classes; D, number of isolates in synthetic groups within the sampling interval classes.*

Supplementary Table 12

Acquired or mutated genes for 12 antibiotic classes per GG (2008-2019, n=4,798, first isolate per person).

	GG	GG0001		GG0005		GG0008		GG0022		GG0030		GG0059		GG0080		GG0398		GG1045		GG2045		Other GGs		All GGs	
	N	**250**		**620**		**554**		**507**		**284**		**106**		**69**		**1516**		**103**		**114**		**675**		**4,798**	
**Antibiotic class**	**Gene**	***n***	**%**	***n***	**%**	***n***	**%**	***n***	**%**	***n***	**%**	***n***	**%**	***n***	**%**	***n***	**%**	***n***	**%**	***n***	**%**	***n***	**%**	***n***	**%**
Aminoglycoside	*aac(6')-aph(2'')*	45	18	24	4	62	11	83	16	12	4	6	6			278	18	75	73			147	22	732	15
	aadD	8	3	66	11	10	2	20	4	41	14	3	3			152	10			2	2	96	14	398	8
	ant(6)-Ia	168	67	4	1	4	1					64	60	46	67	46	3					5	1	337	7
	ant(9)-Ia			92	15	18	3			18	6					278	18			10	9	3	0.4	419	9
	aph(2'')-Ia			2	0.3	1	0.2									8	1					19	3	30	1
	aph(3')-III	201	80	23	4	116	21			66	23	71	67	46	67	4	0.3					123	18	650	14
	str	1	0.4	3	0.5											105	7					1	0.1	110	2
Beta-lactam	*blaZ*	236	94	552	89	512	92	485	96	270	95	89	84	60	87	1,475	97	101	98	106	93	546	81	4,432	92
	mecA	250	100	615	99	553	99.8	506	99.8	283	99.6	106	100	69	100	1,513	99.8	103	100	114	100	618	92	4,730	99
	mecC																					56	8	56	1
Quinolones	*grlA, grlB, gyrA* mut	13	5	263	42	241	44	212	42	49	17	9	8	4	6	333	22	101	98	41	36	152	23	1,418	30
Fosfomycin	*fosB*	1	0.4			1	0.2	1	0.2													1	0.1	4	0.1
Fusidic acid	*fusA* mut			13	2	16	3	9	2	4	1	4	4			8	1	4	4	4	4	7	1	69	1
	fusB			7	1	10	2			1	0.4	1	1	56	81					1	1			76	2
	fusC	51	20	152	25	21	4	2	0.4	6	2	5	5			1	0.1			19	17	129	19	386	8
MLSB	*erm(A)*			92	15	16	3			20	7					173	11			10	9	3	0.4	314	7
	erm(B)			3	0.5	1	0.2					72	68			94	6					25	4	195	4
	erm(C)	163	65	74	12	100	18	106	21	9	3	5	5	20	29	242	16	29	28	4	4	66	10	818	17
	erm(T)															40	3							40	1
	lnu(A)	2	1	2	0.3	5	1	7	1	3	1	3	3			14	1					28	4	64	1
	lnu(B)															111	7					1	0.1	112	2
	lnu(G)	1	0.4													25	2							26	1
	lsa(A)			1	0.2											7	0.5							8	0.2
	lsa(E)															113	7					1	0.1	114	2
	mph(C)	6	2	36	6	159	29			66	23	3	3									97	14	367	8
	msr(A)	7	3	39	6	162	29	1	0.2	67	24	4	4			1	0.1					106	16	387	8
	vga(A)															2	0.1							2	0.04
	vga(A)LC															2	0.1							2	0.04
	vga(A)V					3	1									39	3					3	0.4	45	1
	vga(E)															136	9							136	3
Mupirocin	*ileS* mut			1	0.2	7	1									1	0.1					1	0.1	10	0.2
	mupA			4	1	4	1	1	0.2	3	1	1	1							1	1	1	0.1	15	0.3
Oxazolidone	*cfr*															7	0.5							7	0.1
	optrA															1	0.1							1	0.02
	poxtA															1	0.1							1	0.02
Phenicol	*cat*											45	42			1	0.1					1	0.1	47	1
	cat(pC221)	2	1	5	1	5	1	2	0.4			1	1			4	0.3					17	3	36	1
	cat(pC233)											45	42			1	0.1					1	0.1	47	1
	fexA			48	8	1	0.2					1	1			196	13					1	0.1	247	5
	fexB					1	0.2									1	0.1							2	0.04
Rifampicin	*rpoB* mut			7	1	12	2	3	1	2	1	1	1					2	2	3	3	2	0.3	32	1
Tetracycline	*tet(K)*	164	66	49	8	46	8	9	2	65	23	57	54	39	57	1,091	72	77	75	2	2	174	26	1,773	37
	tet(L)	32	13	7	1	2	0.4			1	0.4					129	9					10	1	181	4
	tet(M)	1	0.4	47	8	17	3			2	1					1,358	90	3	3			8	1	1,436	30
Trimethoprim	*dfrB* mut			5	1			4	1							1	0.1	7	7	3	3	17	3	37	1
	dfrD															1	0.1					1	0.1	2	0.
	dfrE			2	0.3	1	0.2									8	1					19	3	30	1
	dfrG	6	2	56	9	75	14	3	1	54	19					361	24	1	1			103	15	659	14
	dfrK			3	0.5											498	33					6	1	507	11

MLSB, macrolide, lincosamide, streptogramin B. N, number of isolates per GG. n and %, number and percentage of isolates with resistance gene per GG; Mut, resistance due to mutations in gene. On the Top10 GGs are displayed in detail.

Supplementary Figure 8

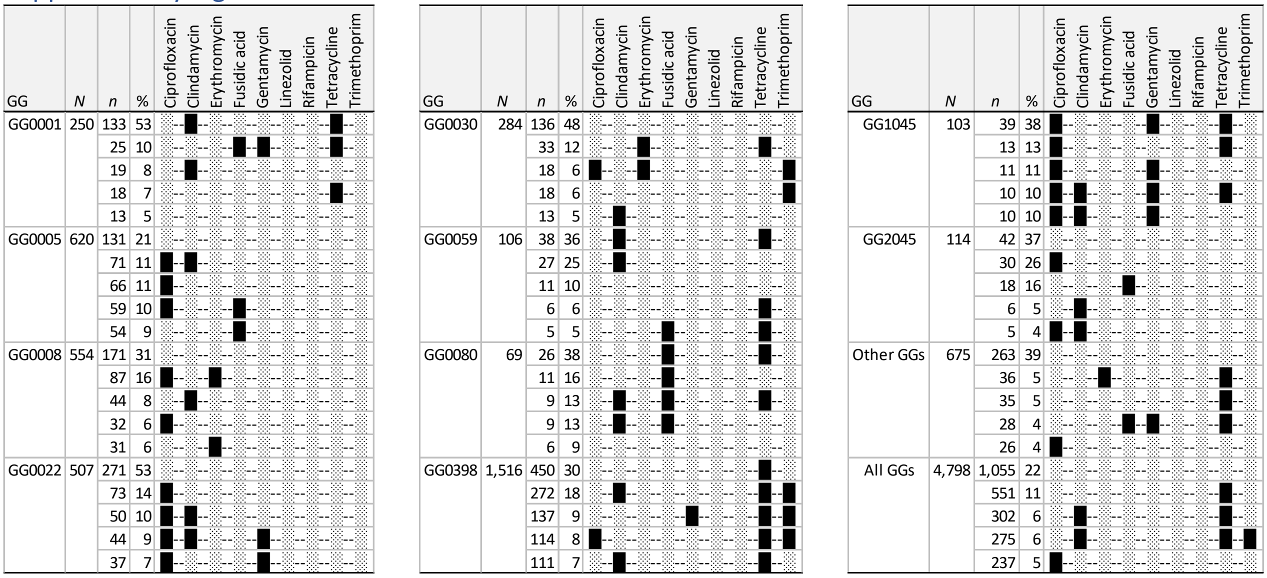

***In silico* resistance profiles for nine antibiotics relevant for the Dutch situation (2008-2019, n=4,798, first isolate per person).** *Resistance against antibiotics is indicated by █ and sensitivity by ░. Only the five most frequently found resistance profiles per GG are displayed. Predicted antibiotic resistance is based on the presence of acquired resistance genes or mutations [DTU]. N, number of isolates per GG; n and %, number and percentage of isolates with predicted resistance per GG. Only the Top10 GGs are displayed in detail.*

Supplementary Table 13

Simultaneous resistance per isolate for nine antibiotics relevant for the Dutch situation, stratified by GG (2008-2019, n=4,798, first isolate per person).

GG	GG0001		GG0005		GG0008		GG0022		GG0030		GG0059		GG0080		GG0398		GG1045		GG2045		Other GGs		All Ggs	
N	250		620		554		507		284		106		69		1516		103		114		675		4798	
Number of resistances	*n*	%	*n*	%	*n*	%	*n*	%	*n*	%	*n*	%	*n*	%	*n*	%	*n*	%	*n*	%	*n*	%	*n*	%
0	13	5	131	21	171	31	271	53	136	48	11	10	6	9	10	1	1	1	42	37	263	39	1,055	22
1	48	19	228	37	143	26	93	18	51	18	39	37	17	25	457	30	1	1	57	50	115	17	1,249	26
>1	189	76	261	42	240	43	143	28	97	34	56	53	46	67	1,049	69	101	98	15	13	297	44	2,494	52
>2	43	17	64	10	104	19	53	10	38	13	8	8	10	14	790	52	75	73			172	25	1,357	28
>3	13	5	10	2	39	7			7	2	3	3			221	15	18	17			100	15	411	9
5			3	0.5	17	3			1	0.4					33	2					11	2	65	1

N, number of isolates per GG. n and %, number and percentage of isolates with resistances per GG.

Supplementary Table 14

Immune evasion cluster typing (IEC typing) of 4,798 isolates.

	GG	GG0001		GG0005		GG0008		GG0022		GG0030		GG0059		GG0080		GG0398		GG1045		GG2045		Other GGs		All GGs	
Genes	*N*	250		620		554		507		284		106		69		1,516		103		114		675		4,798	
sea sep sak chp scn	IEC type	*n*	%	*n*	%	*n*	%	*n*	%	*n*	%	*n*	%	*n*	%	*n*	%	*n*	%	*n*	%	*n*	%	*n*	%
■ ░ ■ ■ ■	A			5	1	2	0.4	32	6	19	7	2	2			1	0.1			1	1	2	0.3	64	1
░ ░ ■ ■ ■	B	3	1	166	27	412	74	398	79	215	76	13	12	2	3	152	10	101	98	96	84	101	15	1,659	35
░ ░ ░ ■ ■	C							3	1			81	76			3	0.2			4	4	25	4	116	2
■ ░ ■ ░ ■	D	70	28	28	5	37	7			1	0.4	2	2			2	0.1					116	17	256	5
░ ░ ■ ░ ■	E	151	60	50	8	25	5	18	4	44	15			64	93	18	1			1	1	217	32	588	12
░ ■ ■ ■ ■	F	1	0.4	128	21					1	0.4					2	0.1					38	6	170	4
░ ■ ■ ░ ■	G			137	22	4	1					4	4			2	0.1					17	3	164	3
Other combinations		3	1	2	0.3	4	1	5	1			4	4	1	1	8	1			6	5	67	10	100	2
No IEC genes		22	9	104	17	70	13	51	10	4	1			2	3	1,328	88	2	2	6	5	92	14	1,681	35

Supplementary Table 15

Distinct PVL bacteriophages in the various GGs in 1,292 isolates (first isolate per person).

		lukF+ and *int+*		att pair	NCBI acc. num.
**GG**	***N***	***n***	**%**		**Closest match**
GG0001	43	42	98	attR - attR	MK940809
GG0005	118	28	24	attL - attR	ON989481
GG0006	7	6	86	attR - attR	MK940809
GG0008	389	388	99.7	attR - attR	MK940809
GG0022	102	100	98	attL - attR	AP025177
GG0030	221	218	99	attL - attR	NC_055047
GG0059	70	67	96	attL - attR	CP039448
GG0072	6	6	100	attR - attR	MG029513
GG0080*	57	57	100	attL - attR	OP661216
GG0088	45	45	100	attR - attR	NC_055048
GG0093	8	8	100	attR - attR	MG029517
GG0121	13	0	0		
GG0152	41	41	100	attR - attR	LN854556
GG0361	1	1	100		
GG0398	111	111	100	attR - attR	MG029517
GG0772	44	43	98	attL - attR	NC_025460
GG1153	2	2	100	attR - attR	MK940809
Total	1,292	1,106	86		

*N, number of isolates per GG. n and %, number and percentage of isolates carrying both the integrase gene (int) and the lukF-PV gene. att, attachment sequences for bacteriophage. The last column shows the NCBI database entry that had the closest match with the fully assembled phage genomes of the various GGs. *, some GG0080 isolates had attL – attR flanking sequences.*

Supplementary Figure 9

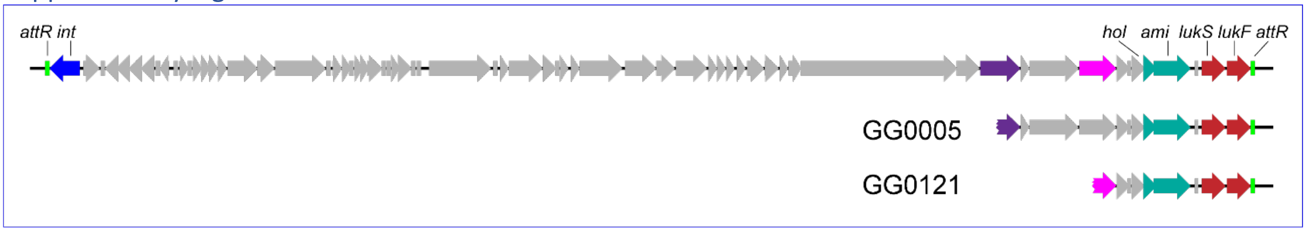

Comparison of the maps of the intact phage genome (phiSa2wa-st5, ON989481) and the truncated GG0005 and GG0121 phage genomes. *Only the attachment sites (attR), the integrase (int), lysis (hol and ami) and PVL-encoding genes (lukS and lukF) are named.*

Supplementary Table 16

Temporal changes in the prevalence of GGs and increasing frequency of PVL-positive isolates.

GG			2008-2010	2011-2013	2014-2016	2017-2019		2008-2019
GG0001	All	*N*	94	174	330	556		1,154
	PVL-positive	*n*	6	7	84	82		179
		%	6	4	25	15		16
GG0005	All	*N*	984	1,092	1,177	1,172		4,425
	PVL-positive	*n*	58	101	126	180		465
		%	6	9	11	15		11
GG0008	All	*N*	1,041	1,132	1,067	1,078		4,318
	PVL-positive	*n*	417	653	644	738		2,452
		%	40	58	60	68		57
GG0022	All	*N*	415	482	878	1,073		2,848
	PVL-positive	*n*	39	95	232	184		550
		%	9	20	26	17		19
GG0030	All	*N*	292	371	423	522		1,608
	PVL-positive	*n*	195	297	359	403		1,254
		%	67	80	85	77		78
GG0059	All	*N*	142	134	198	192		666
	PVL-positive	*n*	66	77	106	108		357
		%	46	57	54	56		54
GG0080	All	*N*	170	111	129	147		557
	PVL-positive	*n*	157	107	118	131		513
		%	92	96	91	89		92
GG0398	All	*N*	3,365	3,080	2,418	2,335		11,198
	PVL-positive	*n*	3	6	30	142		181
		%	0.1	0.2	1	6		2
GG1045	All	*N*	45	408	521	220		1,194
	PVL-positive	*n*		1	2			3
		%		0.2	0.4			0.3
GG2045	All	*N*	363	232	269	199		1,063
	PVL-positive	*n*		5	5	1		11
		%		2	2	1		1
Other GGs	All	*N*	314	452	765	1,458		2,989
	PVL-positive	*n*	105	142	183	324		754
		%	33	31	24	22		25
All GGs	All	*N*	7,225	7,668	8,175	8,952		32,020
	PVL-positive	*n*	1,046	1,491	1,889	2,293		6,719
		%	14	19	23	26		21

Supplementary Figure 10

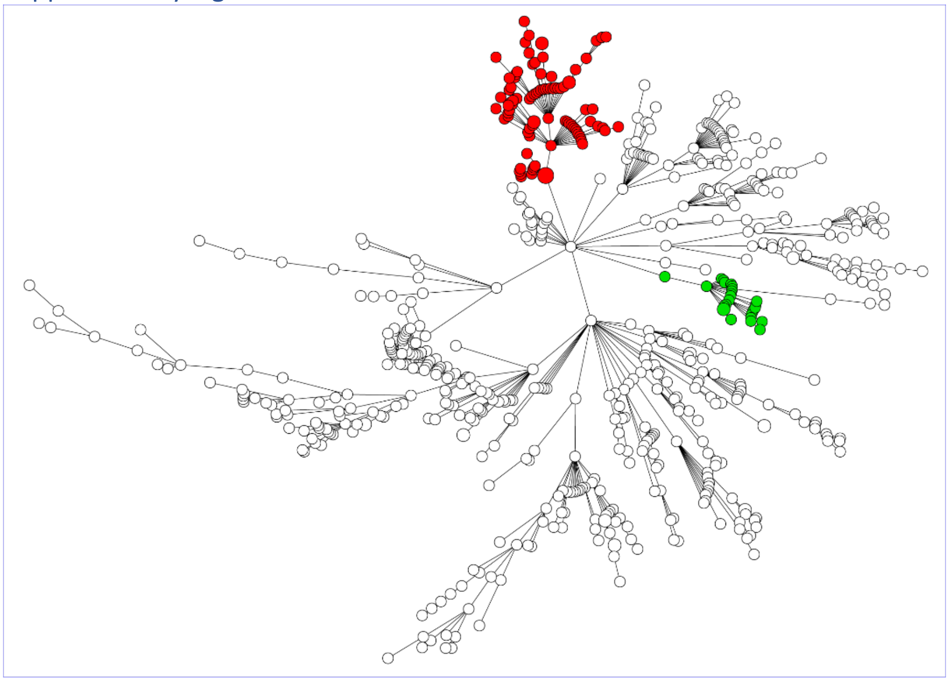

Distribution of PVL-positives in the wgMLST tree of GG0005 isolates (n=620, (first isolate per person). *In green the isolates carrying both the integrase gene and lukF-PV gene (n=28) and in red the lukF-PV carrying isolates without the integrase gene (truncated phage, n=90).*

Supplementary Figure 11

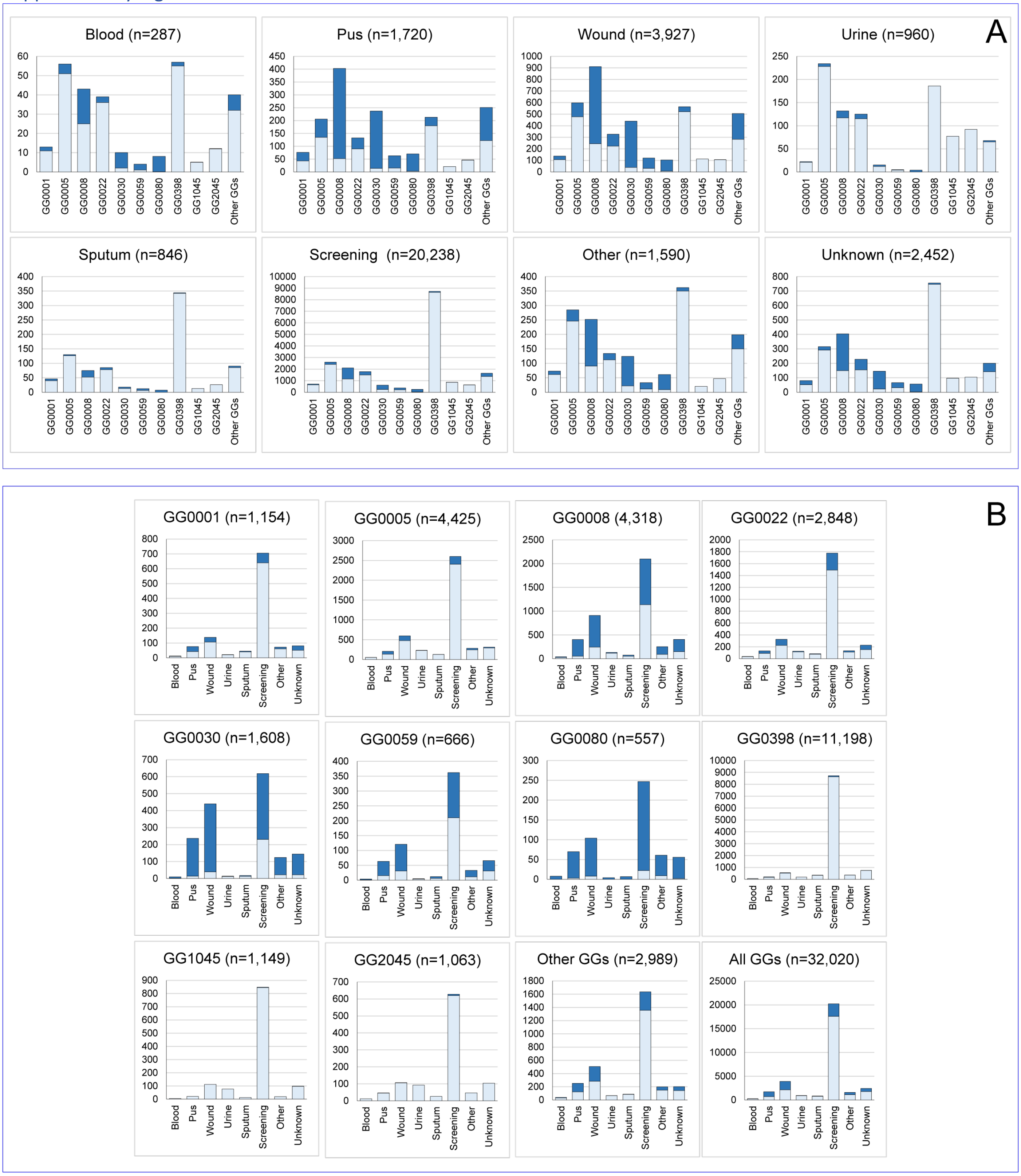

Relationship between specimen types, GGs and presence of PVL-encoding genes in 23,020 isolates with experimental or hypothetical GGs (first isolate per person). *The stacked dark blue bars represent the PVL-positive and the light blue bars the PVL-negative isolates. Panel A shows the distribution of GGs per specimen type. Panel B shows the distribution of specimen types per GG. obtained from the various specimen types and their PVL status. The Y-axes in panel A and B indicate the number of isolates.*

Supplementary Table 17

Mean distances between residential locations of persons carrying PVL-positive and PVL-negative MRSA and the city of The Hague per GG.

	GG	GG0001	GG0005	GG0008	GG0022	GG0030	GG0059	GG0080	GG0398	Other GGs	All GGs
n	PVL-	466	959	326	867	116	78	16	2,166	1,097	6,497
	PVL+	81	177	644	180	374	106	129	140	317	2,149
Mean	PVL-	63	77	82	81	79	70	72	99	72	82
	PVL+	64	64	64	63	61	64	72	62	65	64
	Difference ± SEM	0.2 ± 5	13 ± 4	18 ± 3	18 ± 4	18 ± 5	6 ± 6	0.2 ± 12	37 ± 3	7 ± 3	18 ± 1
	P value t test	0.9707	0.0005	<0.0001	<0.0001	<0.0001	0.292	0.9895	<0.0001	0.0116	<0.0001
	P value F test	0.0224	0.1626	<0.0001	0.1956	0.2199	0.0413	0.7197	<0.0001	0.846	0.2278

n, number of PVL-negative or PVL-positive isolates per GG. SEM, standard error of the mean.

Supplementary Table 18

Resistance genes and resistance mutations in PVL-positive (n=111) and PVL-negative (n=1,405) GG0398 isolates (first isolate per person).

		***lukF*+ (*n*=111)**		***lukF*- (*n*=1,405)**	
**Antibiotic class**	**Gene**	***n***	**%**	***n***	**%**
Aminoglycoside	*aac(6')-aph(2'')*	3	3	275	20
	aadD	1	1	151	11
	ant(6)-Ia			46	3
	ant(9)-Ia	103	93	175	12
	aph(2'')-Ia	3	3	5	0.4
	aph(3')-III			4	0.3
	str			105	7
Beta-lactam	*blaZ*	104	94	1,371	98
	mecA	111	100	1,402	99.8
Ciprofloxacin	*grlA, grlB, gyrA*	3	3	330	23
Fusidic_acid	*fusA*			8	1
	fusC			1	0.1
MLSB	*erm(A)*			173	12
	erm(B)	3	3	91	6
	erm(C)	5	5	237	17
	erm(T)			40	3
	lnu(A)	2	2	12	1
	lnu(B)			111	8
	lnu(G)			25	2
	lsa(A)			7	0.5
	lsa(E)			113	8
	msr(A)			1	0.1
	vga(A)			2	0.1
	vga(A)LC			2	0.1
	vga(A)V			39	3
	vga(E)			136	10
Mupirocin	*ileS*			1	0.1
Oxazolidone	*cfr*			7	0.5
	optrA			1	0.1
	poxtA			1	0.1
Phenicol	*cat*			1	0.1
	cat(pC221)	2	2	2	0.1
	cat(pC233)			1	0.1
	fexA			196	14
	fexB			1	0.1
Tetracycline	*tet(K)*	98	88	993	71
	tet(L)			129	9
	tet(M)			1,358	97
Trimethoprim	*dfrB*			1	0.1
	dfrD			1	0.1
	dfrE	3	3	5	0.4
	dfrG	2	2	359	26
	dfrK			498	35

			***lukF*+ (*n*=111)**		***lukF*- (*n*=1,405)**	
**Gene**	**Allele**	**Gene variant**	***n***	**%**	***n***	**%**
ant(9)-Ia	*ant(9)-Ia_A001*	*ant(9)-Ia_RF_01*			171	12
	ant(9)-Ia_A003	*ant(9)-Ia_NL_01*	103	93		
	Other				3	0.2
blaZ	*blaZ_A007*	*blaZ_RF_007*	103	93	1	0.1
	blaZ_A126	*blaZ_NL_01*			1,009	72
	Other		1	1	360	26

lukF+, PVL-positive; lukF-, PVL-negative. Left, all resistance genes. Right, genes, allelic variants and gene variants for ant(9)-Ia and blaZ. n and %, number and percentage of isolates carrying gene.
