# Supplementary material for: Molecular characterization of MRSA collected during national surveillance between 2008 and 2019 in the Netherlands
Source: Commun Med (Lond). 2023 Sep 12;3:123. doi: 10.1038/s43856-023-00348-z (PMC10497500; doi:10.1038/s43856-023-00348-z)
Supplement: Supplementary file 7 — Description of Additional Supplementary Files [file 43856_2023_348_MOESM7_ESM.pdf]

Description of Additional Supplementary Files

File name: Supplementary Data 1

Description: Details on acquired resistance gene variants and resistance mutations among 4,798 MRSA isolates isolated from humans in the Netherlands 2008-2019.

File name: Supplementary Data 2

Description: Details on virulence gene variants among 4,798 MRSA isolates isolated from humans in the Netherlands 2008-2019.

File name: Supplementary Data 3

Description: Genetic distances between multiple MRSA isolates obtained from the same person (345 isolates from 152 persons).

File name: Supplementary Data 4

Description: Relationship between GGs, MLVA types, CCs and Sequence types of 4,798 MRSA isolates isolated from humans in the Netherlands 2008-2019.
